# Sotatercept analog suppresses inflammation to reverse experimental pulmonary arterial hypertension

**DOI:** 10.1038/s41598-022-11435-x

**Published:** 2022-05-12

**Authors:** Sachindra R. Joshi, Jun Liu, Troy Bloom, Elif Karaca Atabay, Tzu-Hsing Kuo, Michael Lee, Elitza Belcheva, Matthew Spaits, Rosa Grenha, Michelle C. Maguire, Jeffrey L. Frost, Kathryn Wang, Steven D. Briscoe, Mark J. Alexander, Brantley R. Herrin, Roselyne Castonguay, R. Scott Pearsall, Patrick Andre, Paul B. Yu, Ravindra Kumar, Gang Li

**Affiliations:** 1 Discovery Group, Acceleron Pharma Inc., a subsidiary of Merck & Co., Inc., Kenilworth, NJ USA; 2 Division of Cardiovascular Medicine, Department of Medicine, Brigham and Women’s Hospital, Harvard Medical School, Boston, MA 02115 USA; 3 Present Address: Ultivue, Cambridge, MA USA; 4 Present Address: Cellarity, Cambridge, MA USA

**Keywords:** Drug discovery, Cardiology, Diseases, Molecular medicine, Pathogenesis

## Abstract

Sotatercept is an activin receptor type IIA-Fc (ActRIIA-Fc) fusion protein that improves cardiopulmonary function in patients with pulmonary arterial hypertension (PAH) by selectively trapping activins and growth differentiation factors. However, the cellular and molecular mechanisms of ActRIIA-Fc action are incompletely understood. Here, we determined through genome-wide expression profiling that inflammatory and immune responses are prominently upregulated in the lungs of a Sugen-hypoxia rat model of severe angio-obliterative PAH, concordant with profiles observed in PAH patients. Therapeutic treatment with ActRIIA-Fc—but not with a vasodilator—strikingly reversed proinflammatory and proliferative gene expression profiles and normalized macrophage infiltration in diseased rodent lungs. Furthermore, ActRIIA-Fc normalized pulmonary macrophage infiltration and corrected cardiopulmonary structure and function in *Bmpr2* haploinsufficient mice subjected to hypoxia, a model of heritable PAH. Three high-affinity ligands of ActRIIA-Fc each induced macrophage activation in vitro, and their combined immunoneutralization in PAH rats produced cardiopulmonary benefits comparable to those elicited by ActRIIA-Fc. Our results in complementary experimental and genetic models of PAH reveal therapeutic anti-inflammatory activities of ActRIIA-Fc that, together with its known anti-proliferative effects on vascular cell types, could underlie clinical activity of sotatercept as either monotherapy or add-on to current PAH therapies.

## Introduction

PAH is a severe progressive disease characterized by obliterative vascular remodeling and increased resistance in the pulmonary circulation leading to right ventricle (RV) hypertrophy, right heart failure, and premature death. Major factors that contribute to the complex etiology of PAH pathogenesis include genetic mutations, pulmonary inflammation, systemic immune dysregulation, imbalanced pulmonary vascular proliferation and apoptosis, biomechanical disturbances, and potentially enhanced endothelial-mesenchymal transition (EndMT)^1–6^. Pulmonary vascular remodeling and functional impairments are considerably advanced by the time PAH is typically diagnosed^7^, rendering therapeutic treatment especially challenging for this condition. Currently approved PAH therapies improve symptoms and functional capacity, but they do not directly target vascular or RV remodeling^4^. Thus, there is an urgent need for mechanistically distinct therapies that target cardiopulmonary remodeling^8^ to reverse PAH disease progression and improve outcomes.

Signaling by ligands of the transforming growth factor-β (TGF-β) superfamily is heavily implicated in PAH pathogenesis. Diverse loss-of-function mutations associated with heritable PAH have been identified in genes encoding bone morphogenetic protein (BMP) signaling molecules and their downstream effectors, including *BMPR2*, *ACVRL1*, *ENG*, *GDF2* (encoding BMP9), and *SMAD9* (SMAD8), implying that the BMP signaling branch of the superfamily exerts a protective function that is compromised in disease^3,9^. BMP receptor type II (BMPRII) mediates signaling by multiple BMPs, notably including circulating BMP9 and BMP10, and signals through SMAD1/5/8 intracellularly. This receptor is a particularly important regulator of vascular homeostasis and serves a critical gate-keeping function in PAH^10^. Consistent with the anti-inflammatory role of BMPRII in pulmonary endothelial cells^11–13^, inflammation has been implicated as a likely second hit required to induce severe vascular pathology in the context of reduced BMPRII signaling^14^.

In contrast to the protective function afforded by vascular SMAD1/5/8 signaling, multiple lines of evidence indicate that excessive activation of the SMAD2/3 branch is pathogenic in PAH^15,16^ as well as in vascular disease broadly^17–20^. TGF-β is considered a prototypical SMAD2/3 pathway–activating ligand and a master homeostatic regulator of the respiratory system, with roles in inflammatory and immune regulation^21^ as well as PAH pathogenesis^15,16^. Functional antagonism between SMAD1/5/8 and SMAD2/3 signaling pathways at multiple levels and under diverse pathologic conditions^22,23^ supports the hypothesis that imbalance between these two superfamily branches is central to PAH pathology^15,16,24^. However, despite compelling evidence of SMAD2/3 involvement in PAH, few studies have implicated SMAD2/3-pathway ligands other than TGF-β in this disease^25–29^. These insights raise the possibility that multiple SMAD2/3 pathway–activating ligands drive pathologic vascular remodeling in PAH, but their identities, respective contributions, and cellular sites of action remain incompletely characterized.

Sotatercept and its rodent analog, RAP-011, are recombinant ActRIIA-Fc fusion proteins capable of sequestering multiple activin-class ligands—including activin A, activin B, growth differentiation factor 8 (GDF8) and GDF11—that preferentially activate the SMAD2/3 pathway^23,28^. In a phase 2 trial, sotatercept significantly improved pulmonary vascular resistance in patients with PAH receiving background therapy (NCT03496207)^30^, and this agent is the focus of ongoing clinical investigation (NCT03738150, NCT04576988, NCT04811092, NCT04896008). We recently described elevated expression of activin A, GDF8, and GDF11 in lung lesions from PAH patients and rodent models of PH, together with robust anti-proliferative and pro-apoptotic activity of ActRIIA-Fc in cellular and preclinical animal models of PAH^28^. Activin-class ligands, particularly activin A, promote inflammatory processes in some disease contexts^31–33^ but have not been linked with pulmonary vascular inflammation in PAH^28,29^.

To better understand the mechanism by which ActRIIA-Fc exerts vascular anti-remodeling effects, in the present study we used RNA-seq and pathway analyses to determine the impact of ActRIIA-Fc therapy on the pulmonary gene signature in severe experimental PAH and to determine the degree to which this model of severe angio-obliterative PAH resembles the gene signature in PAH patients. Additionally, we explored the potential activities and immune effects of ActRIIA-Fc treatment in a model of heritable PAH arising from *Bmpr2* haploinsufficiency. We also investigated the contribution of individual activin-class ligands to macrophage activation in vitro and the importance of multi-ligand sequestration for cardiopulmonary effects of ActRIIA-Fc in vivo. Finally, we sought to determine whether ActRIIA-Fc maintains its beneficial activity in severe experimental PAH when used in therapeutic combination with a vasodilator and whether therapeutic effects of ActRIIA-Fc in severe experimental PAH persist after treatment cessation as an indication of potential disease modification.

## Results

### Expression profiling and pathway analysis in a rat model of severe angio-obliterative PAH

**Figure 1:**
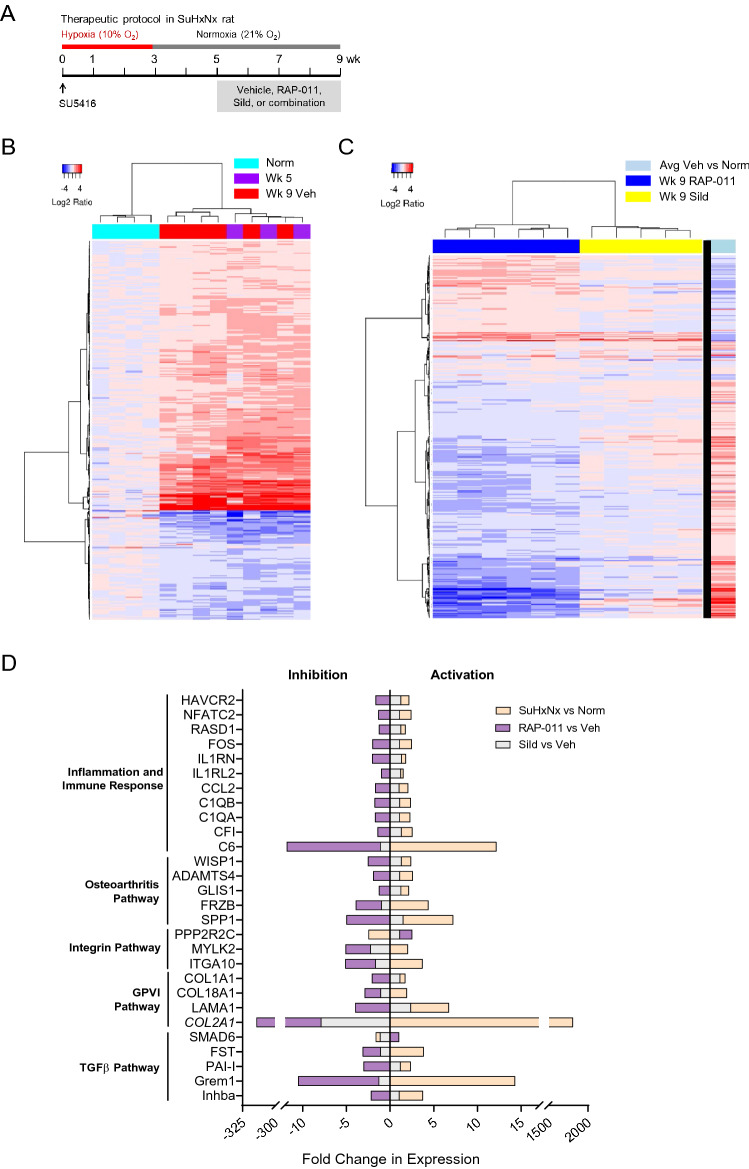
Therapeutic treatment with ActRIIA-Fc broadly normalizes pulmonary gene expression in severe experimental PAH. (**A**) Experimental approach used to evaluate therapeutic effects of RAP-011 in a Sugen-hypoxia-normoxia (SuHxNx) rat model of severe PAH. Rats were treated on day 0 with a single dose of SU5416 (20 mg/kg) and exposed to normobaric hypoxia (10% O_2_) for 3 weeks followed by 6 weeks of normoxia to allow disease progression. Rats were additionally treated with RAP-011 (2.5 mg/kg, s.c., twice weekly), sildenafil (30 mg/kg, p.o., twice daily), combination therapy with RAP-011 and sildenafil, or vehicle (PBS) for 4 weeks starting on week 5 post SU5416. (**B**) Heat map of differentially expressed genes (DEGs) in lung from untreated SuHxNx rats at week 5 (Wk 5) and vehicle-treated SuHxNx rats at week 9 (Wk 9 Veh), each compared to normal (Norm). Genes were clustered using the Ward method. (**C**) Heat map of DEGs at week 9 in lung from SuHxNx rats treated with RAP-011 or sildenafil (Sild), each compared to a normalized average from vehicle-treated SuHxNx rats at week 9 (right column). (**D**) IPA-based classification of selected genes exhibiting significant differential expression at week 9 in lung from SuHxNx rats treated with vehicle, RAP-011, or sildenafil.

To investigate the mechanistic bases for effects of RAP-011 reported previously in experimental PH^28^, we conducted RNA expression profiling and pathway analysis of lung tissue in a Sugen-hypoxia-normoxia (SuHxNx) rat model of severe angio-obliterative PAH (Fig. 1A, Supplemental Fig. 1). This model mimics important features of human PAH, including pathologic pulmonary vascular remodeling, perivascular pulmonary inflammation, marked RV dysfunction, and a progressive course culminating in severe occlusive arteriopathy^34,35^. Moreover, therapeutic effects observed in this preclinical model—with the normoxic progression phase included—are considered broadly predictive of therapeutic efficacy in patients^36^.

To set the stage for expression profiling in lung, we confirmed that hemodynamic parameters including RV systolic pressure (RVSP) and total pulmonary resistance index (TPRI) were significantly elevated in untreated SuHxNx rats at the onset of therapeutic treatment (week 5) and continued through week 9 (Supplemental Fig. 1). Delayed treatment with RAP-011 starting at week 5 markedly improved (reversed) hemodynamic deficits by week 9 compared to vehicle-treated SuHxNx rats (Supplemental Fig. 1). We confirmed that RAP-011 treatment produced significantly greater improvement than sildenafil (Supplemental Fig. 1), a conventional vasodilatory agent commonly used as first or second line therapy in PAH. Pulmonary histology in untreated week 5 and vehicle-treated week 9 SuHxNx rats revealed significantly elevated abundance of remodeled and occluded arteries, consistent with present hemodynamic results (Supplemental Fig. 1), and confirmed our previous observations that RAP-011 treatment causes regression of vascular remodeling more effectively than therapy with a standard vasodilator^28^.

We next identified genes differentially expressed in SuHxNx rat lung compared with normal rat lung. Using hierarchical cluster analysis of RNA-seq data, we identified 345 differentially expressed genes (DEGs) with a fold-change ≥ 1.5 and adjusted p-value ≤ 0.05 at both week 5 and week 9. Of these 345 DEGs, 248 were upregulated and 97 were downregulated in diseased rat lung compared with normal tissue (Fig. 1B). Therapeutic treatment of SuHxNx rats with RAP-011 from week 5 to week 9 exerted a robust normalizing effect on this pathologic gene-expression profile (Fig. 1C). By week 9, RAP-011 treatment normalized expression of 207 out of 248 (84%) upregulated DEGs and 69 out of 97 (71%) downregulated DEGs. In contrast, therapeutic treatment of SuHxNx rats with sildenafil altered expression of only 27 of 345 (8%) total DEGs (Fig. 1C). Principal component analysis revealed that lung tissue from RAP-011-treated SuHxNx rats exhibited a gene expression profile globally resembling normal tissue, whereas the profile for sildenafil-treated SuHxNx rat lungs more closely resembled that of untreated SuHxNx rat lungs at week 5 (Supplemental Fig. 2). These results indicate that therapeutic treatment with RAP-011 exerts a distinct corrective effect on the global pathologic gene-expression profile in severe experimental PAH unmatched by treatment with a standard vasodilator currently available for PAH therapy.

We then used Ingenuity Pathway Analysis (IPA) to identify dysregulated pathways and potential upstream regulators associated with all DEGs (defined by adjusted p-value < 0.001) based on a comparison of lung tissue in untreated SuHxNx rats at both week 5 and week 9 with lung tissue in normal rats. This analysis identified 58 pathways significantly dysregulated in diseased lungs at both week 5 and week 9 compared with normal tissue. As ranked by Fisher’s method, the top canonical pathways include those mediating endothelial and vessel injury responses (coagulation, prothrombin activation, and glycoprotein VI pathways); inflammation and immune response (complement, dendritic cell maturation, pattern recognition, interleukin-10, and innate and adaptive immune response pathways); and TGF-β signaling (Table 1), all of which have been implicated in PAH progression in either patients or preclinical models, or both^2,4^. Interestingly, the top-ranked upstream regulator identified by this analysis was tumor necrosis factor (TNF), a key regulator of inflammatory and immune responses that also inhibits expression of BMPRII^37^. Other top-ranked upstream regulators, such as VCAN, EPHB1, EGLN1, and TSC2, are known to regulate cell proliferation and migration. Additional candidate upstream regulators identified by IPA include TGFBR2 and genes involved in the mitogen-activated protein kinase pathway (ERK, p38MAPK, and MAP2k1), well-characterized regulators of inflammation and cell proliferation (Table 1). Overall, the pathways and upstream regulators implicated by this analysis in SuHxNx pulmonary pathology depict a phenotype of highly activated inflammation and proliferation.

**Table 1:** Top ranked pathways and upstream regulators by Ingenuity Pathway Analysis in lungs from a SuHxNx rat model of severe PAH.

	*-log10(p*Values)	*z*-Scores
**Pathway**		
Hepatic fibrosis/hepatic stellate cell activation	10.99	–
Complement system	9.68	(1.41, 1.13)
Apelin Liver signaling pathway	8.88	(0.33, 0.82)
Role of osteoblasts, osteoclasts and chondrocytes in rheumatoid arthritis	7.65	–
Granulocyte adhesion and diapedesis	7.58	–
Atherosclerosis signaling	6.03	–
Coagulation system	3.75	(0.816, − 0.48)
Intrinsic prothrombin activation pathway	3.6	(2, 1.34)
Dendritic cell maturation	3.58	(0.26, 2.33)
Role of macrophages, fibroblasts and endothelial cells in rheumatoid arthritis	3.5	–
LXR/RXR activation	3.34	(− 1.6, − 0.38)
Osteoarthritis pathway	3.32	(2, 1)
Cell Cycle: G2/M DNA damage checkpoint regulation	3.15	–
Acute phase response signaling	3.01	(0.71, 1.63)
Role of pattern recognition receptors in recognition of bacteria and viruses	2.94	(0.71, 1)
GP-VI signaling pathway	2.93	(1.51, 1.9)
IL-10 signaling	2.73	–
Communication between innate and adaptive immune cells	2.52	–
STAT3 pathway	2.36	–
Crosstalk between dendritic cells and natural killer cells	2.17	–
TGF-β signaling	2.14	(1.89, 2)
G-protein coupled receptor signaling	2.12	–
Altered T cell and B cell signaling in rheumatoid arthritis	2.1	–
Th1 and Th2 activation pathway	2.04	–
**Upstream regulators**		
TNF	18.96	(1.32, 0.67)
VCAN	11.01	(− 1.85, − 1.98)
EPHB1	10.09	(2.44, 2.22)
ERK1/2	7.49	(1.61, 1.98)
EGLN1	7.06	–
NFAT5	6.43	–
p38MAPK	6.11	(− 0.68, − 0.45)
ERK	6.09	(1.07, –)
MAP2K1	5.35	(0.9, –)
TSC2	5.11	(− 1.73, − 2.33)
Histone h3	5.11	–
TGFBR2	5.06	(2.33, –)
GNA14	4.86	–
NFκB	4.63	(2.21, 1.92)
P2RY2	4.52	–

### Concordance in aberrant gene expression profiles between a rat model of severe angio-obliterative PAH and PAH patients

We next investigated the potential relevance of these dysregulated pathways to PAH pathogenesis in patients. Publicly available, human transcriptome data collected from 58 PAH lungs and 25 control lungs (https://www.ncbi.nlm.nih.gov/geo/query/acc.cgi?acc=GSE117261) were subjected to IPA to identify significantly dysregulated pathways, which were then compared with the pathways identified in SuHxNx rat lung (Table 1). Consistent with published human data, the top-ranked pathways identified here in lung tissue from PAH patients included those involving G protein-coupled receptors and several pathways associated with inflammation and immune responses. Importantly, IPA identified 18 pathways related to inflammatory and immune responses that are significantly dysregulated in lung tissue from both PAH patients and the SuHxNx rat model of severe PAH (Table 2). Among other such pathways are those mediating TGF-β and BMP signaling. These results obtained by IPA indicate concordance in dysregulated gene expression between the SuHxNx rat model and PAH patient lung tissues and provide corroborating evidence that the SuHxNx rat used here is a robust model of human PAH, particularly regarding its exuberant inflammatory gene expression signature.

**Table 2:** Top common pathways in lungs from SuHxNx rats and PAH patients.

Pathway	Human		Rat	
	*− Log*_*10*_* (P v*alue)	*Z*-Score	*− Log*_*10*_* (P v*alue)	*Z*-Score
G-protein coupled receptor signaling	4.95	–	2.12	–
Osteoarthritis pathway	4.65	− 0.229	3.32	(2, 1)
cAMP-mediated signaling	4.12	− 0.426	1.65	(− 1.15, − 0.38)
Hepatic fibrosis/hepatic stellate cell activation	4.06	–	10.99	–
Complement system	4	1	9.68	(1.41, 1.13)
Cardiac hypertrophy signaling	3.89	0.324	1.29	(0.18, − 0.78)
Granulocyte adhesion and diapedesis	3.68	–	7.58	–
Role of pattern recognition receptors in recognition of bacteria and viruses	3.39	− 0.905	2.94	(0.71, 1)
Altered T cell and B cell signaling in rheumatoid arthritis	3.15	–	2.11	–
Axonal guidance signaling	3.11	–	2.78	–
Role of macrophages, fibroblasts and endothelial cells in rheumatoid arthritis	3	–	3.5	–
Th2 pathway	2.8	− 0.707	1.4	(− 1.13, − 0.45)
Role of osteoblasts, osteoclasts and chondrocytes in rheumatoid arthritis	2.33	–	7.65	–
T cell exhaustion signaling pathway	2.25	1.414	1.58	(0.71, 1.34)
LPS/IL-1 mediated inhibition of RXR function	2.11	− 1.134	1.76	(1.89, –)
BMP signaling pathway	2.09	0.333	1.57	(− 1.89, –)
IL-10 signaling	2.08	–	2.73	–
TGF-β signaling	1.31	0.378	2.14	(1.89, 2)

### ActRIIA-Fc targets pulmonary inflammatory markers and macrophage infiltration in diverse models of PH

To assess the effects of therapeutic treatment with either RAP-011 or sildenafil on dysregulated pathways in SuHxNx rat lung, we determined relative expression levels of DEGs associated with these pathways. Therapeutic treatment with RAP-011 reversed the aberrant expression of many genes in lung that were otherwise activated in the SuHxNx disease state (Fig. 1D). Genes differentially expressed as a result of RAP-011 treatment prominently included those in pathways associated with inflammatory and aberrant immune responses (Fig. 1D). Other DEGs are associated with the TGF-β superfamily pathway, most notably *Grem1* (gremlin-1), an endogenous BMP antagonist implicated in endothelial cell proliferation and PAH^38^. Changes in expression associated with RAP-011 treatment were especially pronounced in the cases of *Col2A1*, *C6*, and *Grem1*. In contrast with the striking corrective effects of RAP-011 treatment, therapeutic treatment with sildenafil produced limited changes in the expression of disease-associated genes (Fig. 1D). These results provide a pulmonary gene expression signature corresponding to the potent therapeutic anti-remodeling effects of RAP-011 in this model of severe PAH.

**Figure 2:**
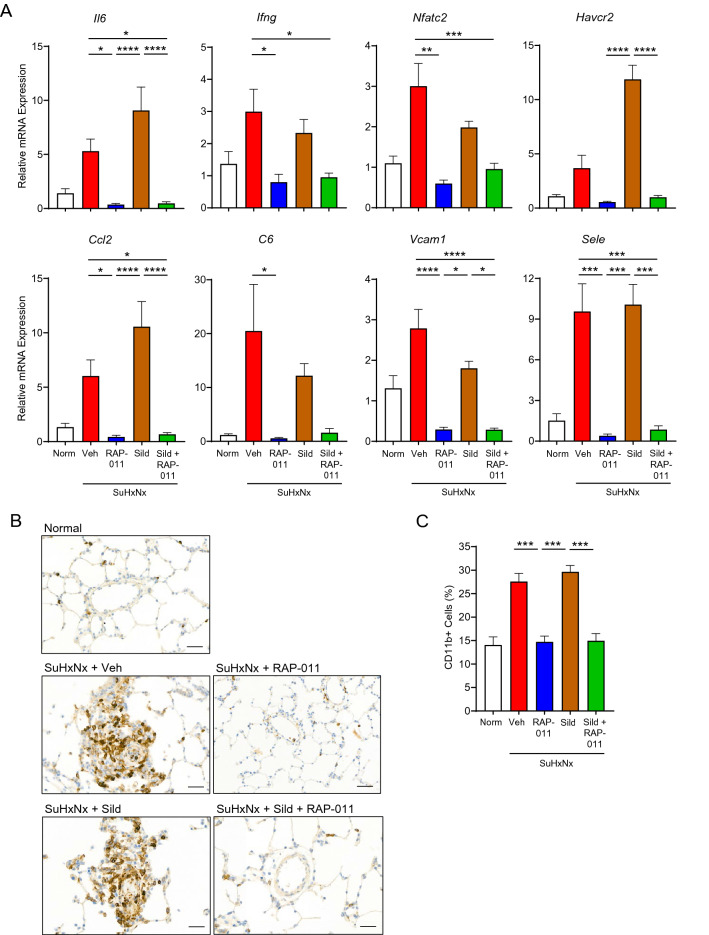
Therapeutic treatment with ActRIIA-Fc suppresses pulmonary inflammation and aberrant immune responses in severe experimental PAH. (**A**) Levels of *Il6*, *Ifng*, *Nfatc2*, *Havcr2*, *Ccl2*, *C6*, *Vcam1*, and *Sele* mRNA in lung of normal rats (Norm) or SuHxNx rats treated with vehicle (Veh), RAP-011, sildenafil (Sild), or a combination of sildenafil and RAP-011. Data are means ± SEM (n = 6–9 rats per group). (**B**) Representative images of lung sections immunostained for macrophage marker CD11b revealing prominent clusters of labeled perivascular cells in severe experimental PAH after treatment with vehicle or sildenafil but not RAP-011. (**C**) Quantification of CD11b-positive cells in lung based on assessment of 40 high-magnification fields per rat. Scale bar, 50 µm. Data are means ± SEM (n = 6–9 rats per group). Analysis by one-way ANOVA and Tukey post hoc test (*P < 0.05, **P < 0.01, ***P < 0.001, ****P < 0.0001).

We then examined the effects of RAP-011 therapy on expression of selected inflammatory and immune molecular markers in SuHxNx rat lung. PAH progression in SuHxNx rats was associated at week 9 with significantly elevated expression of eight key markers, including *Il6* and *Ccl2*, and in each case therapeutic treatment with RAP-011—but not vehicle or sildenafil—fully normalized their mRNA levels (Fig. 2A). Importantly, RAP-011 and sildenafil in combination normalized expression of these markers as effectively as RAP-011 monotherapy (Fig. 2A), indicating that RAP-011 provides robust benefit in this model even when used in combination with a standard vasodilator.

We investigated the effects of therapeutic treatment with RAP-011 on expression of CD11b, a marker for activated alveolar macrophages—the predominant immune cells within alveolar airspaces^39^. As determined by immunostaining, perivascular CD11b + cell abundance was significantly elevated in the lungs of SuHxNx rats at week 9 compared with levels in normal rat lungs (Fig. 2B,C). As with other immune markers, therapeutic treatment with RAP-011 fully blocked pulmonary infiltration of CD11b + cells, whereas treatment with either vehicle or sildenafil had no effect (Fig. 2B,C). RAP-011 fully normalized the number of pulmonary CD11b + cells when administered in combination with sildenafil (Fig. 2B,C), thus demonstrating the anti-inflammatory efficacy of RAP-011 as either monotherapy or add-on therapy in this setting. As determined by fluorescence-activated cell sorting, preventive treatment with RAP-011 similarly blocked pulmonary infiltration of CD11b^+^ cells in monocrotaline-treated rats, another established model of inducible PH^40^, while also preventing development of elevated RVSP and RV hypertrophy (Supplemental Fig. 3). These results demonstrate that therapeutic treatment with ActRIIA-Fc—unlike standard PAH therapy with sildenafil—robustly inhibits inflammation and perivascular monocytic infiltration as important components of its tissue-level anti-remodeling activity in PH models.

### Activin-class ligands contribute to macrophage activation and cardiopulmonary remodeling

**Figure 3:**
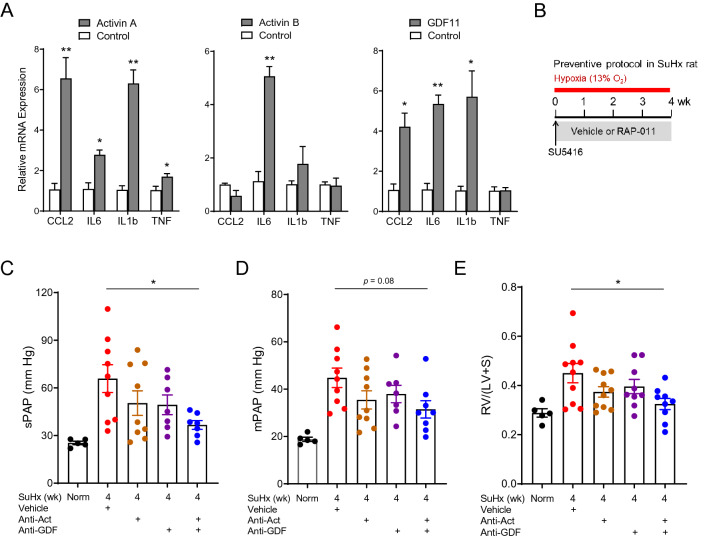
Multiple ligands contribute to macrophage activation in vitro and cardiopulmonary remodeling in a SuHx rat model of PH. (**A**) Expression of markers of macrophage activation in THP-1 monocytes in vitro without treatment (Control) or after treatment with activin A (5 ng/ml), activin B (50 ng/ml), or GDF11 (5 ng/ml). Analysis by unpaired t-test (*P < 0.05, **P < 0.01 vs. control). (**B**) Experimental approach used to test effects of multi-ligand inhibition in a SuHx rat model of PH. Rats were treated with a single dose of SU5416 (20 mg/kg, s.c.), exposed to normobaric hypoxia (13% O_2_), and treated s.c. twice weekly with separate antibodies against activin A and activin B (anti-Act, 10 mg/kg each), an antibody with dual specificity for GDF8 and GDF11 (anti-GDF, 10 mg/kg), combined anti-Act and anti-GDF, or vehicle (PBS) for 4 weeks starting one day post SU5416. (**C**) sPAP, (**D**) mPAP, and (**E**) Fulton index. Data are means ± SEM (n = 5–9 rats per group). Analysis by one-way ANOVA and Tukey post hoc test (*P < 0.05, **P < 0.01, ****P < 0.0001).

We next examined effects of activin-class ligands on expression of molecular markers of inflammatory macrophage activation in vitro. THP1 cells, a human monocytic cell line, exhibited differential patterns of gene induction on exposure to activin A, activin B, or GDF11 (Fig. 3A). Specifically, activin A increased expression of *Ccl2*, *Il6*, *Il1b*, and *Tnf*; activin B selectively increased *Il6*; and GDF11 increased *Ccl2*, *Il6*, and *Il1b*. As expected, treatment of THP1 cells with a triple combination of activin A, activin B, and GDF11 also caused upregulation of inflammatory molecular markers, and co-treatment with a human ActRIIA-Fc analog (ACE-011) prevented this effect (Supplemental Fig. 4). These results indicate that activin-class ligands can individually exert differential activating effects on monocytes to promote a proinflammatory macrophage phenotype in vitro.

We used neutralizing antibodies directed against either activins or GDFs to investigate the respective contribution of these ligands to in vivo cardiopulmonary effects of RAP-011, which binds activins and GDFs with high affinity and exhibits slow off-rates advantageous for ligand sequestration^23^. In a preventive SuHx rat model (Fig. 3B), elevated hemodynamic parameters such as systolic pulmonary artery pressure (sPAP) and mean pulmonary artery pressure (mPAP) were normalized more effectively by dual combination treatment with antibodies directed against activin A/B and GDF8/GDF11 than by separate antibody treatments (Fig. 3C,D). Dual antibody treatment also normalized RV hypertrophy more effectively than separate antibody treatments (Fig. 3E). Although sequestration of either activins or GDFs conveyed partial protection in these experiments, our results imply that sequestration of multiple SMAD2/3-pathway ligands—likely activins, GDF8, and GDF11 in combination—provides a greater spectrum of therapeutic benefit and underlies RAP-011–induced reversal of cardiopulmonary impairments in experimental PH.

### ActRIIA-Fc reverses cardiac remodeling in severe experimental PAH

**Figure 4:**
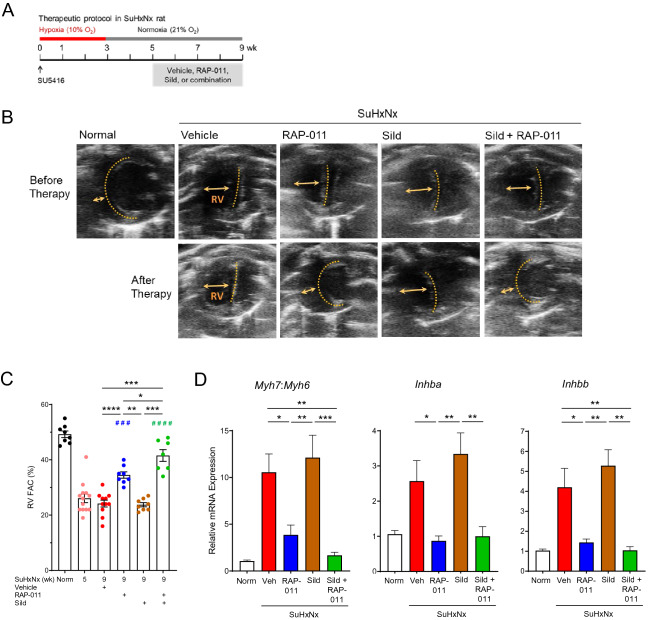
ActRIIA-Fc reverses cardiac remodeling and expression of key cardiac genes in severe experimental PAH. (**A**) Experimental approach used to evaluate therapeutic effects of RAP-011 in the SuHxNx rat model of severe PAH. See Fig. 1 for details. (**B**) Pairs of representative echocardiographic images obtained at the end of diastole from the same SuHxNx rats before and after therapy. (**C**) RV fractional area change (RV FAC). Data are means ± SEM (n = 7–11 rats per group). (**D**) Ratio of myosin heavy-chain isoform expression (*Myh7*:*Myh6*) and levels of *Inhba* and *Inhbb* in the RV of normal or SuHxNx rats. Data are means ± SEM (n = 6–11 rats per group). Analysis by one-way ANOVA and Tukey post hoc test (*P < 0.05, **P < 0.01, ***P < 0.001, ****P < 0.0001; ### P < 0.001 vs. wk 5, #### P < 0.0001 vs. wk 5).

We further investigated the cardioprotective effects of RAP-011 monotherapy and compared them with sildenafil in the SuHxNx model, which exhibits RV hypertrophy and an impaired cardiac index at the onset of therapeutic treatment^28^ (Supplemental Fig. 5). Therapeutic treatment with RAP-011 starting 5 weeks after disease initiation improved these parameters significantly by week 9 and yielded greater improvement than treatment with sildenafil (Supplemental Fig. 5). Serial echocardiography revealed that RAP-011 therapy reversed RV dilatation, septal wall flattening, and RV fractional area change (RVFAC), whereas sildenafil did not (Fig. 4B,C). We confirmed that RAP-011 therapy alleviated abnormalities in pulmonary artery acceleration time (PAAT) and RV free-wall thickness (RVFWT) more effectively than sildenafil (Supplemental Fig. 5).

We examined effects of therapeutic treatment with RAP-011 on selected molecular markers of cardiac dysfunction in the SuHxNx model of severe PAH. Cardiac remodeling and heart failure are associated with increased activin–ActRIIA/B signaling and an expression shift from myosin heavy chain isoform α to isoform β (increased *Myh7*:*Myh6* ratio)^41,42^. As compared with normal heart tissue, RV tissue from vehicle-treated SuHxNx rats at week 9 displayed increased expression of β-subunits for activin A (*Inhba*) and activin B (*Inhbb*) and an increased ratio of *Myh7*:*Myh6* expression (Fig. 4D). In each case, therapeutic treatment with RAP-011 partially or fully normalized expression of these markers of cardiac dysfunction, whereas treatment with either vehicle or sildenafil did not. Moreover, co-treatment of SuHxNx rats with RAP-011 and sildenafil decreased levels of pSmad3 in the RV, and RAP-011 monotherapy was sufficient to increase levels of pSmad1/5/8 in the RV (Supplemental Fig. 6). Together, these results extend our previous findings in a SuHxNx model and confirm that inhibition of multiple activin-class ligands by ActRIIA-Fc reverses aberrant cardiac gene expression, reverses cardiac structural remodeling, and partially corrects an imbalance in RV Smad signaling in severe experimental PAH.

### ActRIIA-Fc is effective when used in combination with a vasodilator in severe experimental PAH

As part of the experiments with SuHxNx rats described above, we investigated whether combined therapy with RAP-011 and sildenafil conferred greater therapeutic benefit for established disease than their respective monotherapies. In comparisons not reported previously, combined therapy with RAP-011 and sildenafil produced significantly greater improvement in cardiac endpoints than treatment with sildenafil alone (Fig. 4B–D; Supplemental Fig. 5). A similar pattern was observed for hemodynamic deficits and vascular occlusion (Supplemental Fig. 1). For some parameters, combination therapy seemed more effective than RAP-011 monotherapy (Fig. 4; Supplemental Figs. 1, 5), although the majority of the benefit was provided by RAP-011. These results demonstrate greater effectiveness of ActRIIA-Fc monotherapy compared to sildenafil monotherapy, as well as ActRIIA-Fc effectiveness as an add-on therapy in this rat model of severe angio-obliterative PAH, consistent with the efficacy observed in PAH patients receiving background therapies^30^. These results further solidify the view that ActRIIA-Fc operates through mechanisms largely distinct from those of current PAH therapies.

### ActRIIA-Fc alleviates cardiopulmonary remodeling and macrophage infiltration in a model of heritable PAH arising from *Bmpr2* haploinsufficiency

**Figure 5:**
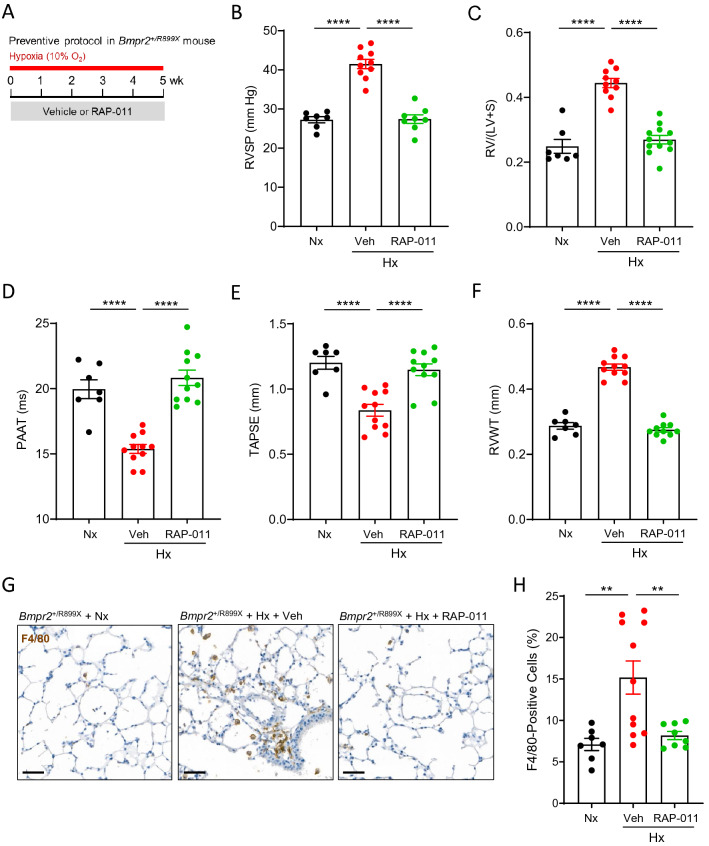
ActRIIA-Fc reduces pulmonary macrophage infiltration and prevents PH in a mouse model of *Bmpr2* haploinsufficiency. (**A**) Experimental approach used to evaluate cardiopulmonary effects of RAP-011 in mice with *Bmpr2* haploinsufficiency. *Bmpr2*^+*/R899X*^ mice were housed under normoxic conditions (Nx) as controls or exposed to normobaric hypoxia (10% O_2_) and treated twice-weekly with either RAP-011 (10 mg/kg, s.c.) or vehicle (PBS) for 5 weeks. (**B**) RVSP, (**C**) Fulton index, (**D**) PAAT, (**E**) TAPSE, and (**F**) RVWT. (**G**) Representative images of lung sections immunostained for macrophage marker F4/80. (**H**) Quantification of F4/80-positive cells in lung based on assessment of 30 high-magnification fields per mouse. Scale bar, 50 µm. Data are means ± SEM (n = 7–10 per group). Analysis by one-way ANOVA and Tukey post hoc test. *P < 0.05, **P < 0.01, ***P < 0.001, ****P < 0.0001.

Loss-of-function mutations in *BMPR2* have been identified in heritable PAH, and even idiopathic forms of PAH are associated with either reduced BMPRII protein expression or diminished BMPRII signaling^3,43^. Therefore, we generated *Bmpr2* haploinsufficient mice as reported by others^44^ and evaluated RAP-011 activity in these mutant mice under hypoxic conditions (Fig. 5A). Analysis of genomic DNA confirmed that *Bmpr2*^+*/R899X*^ mice possess a heterozygous nucleotide substitution at the expected position, and immunoblotting confirmed reduced levels of BMPRII protein in lung lysates consistent with a truncated protein product and/or nonsense-mediated mRNA degradation (Supplemental Fig. 7).

Exposure of *Bmpr2*^+*/R899X*^ mice to hypoxia elevated RVSP, induced RV hypertrophy, and produced abnormalities in PAAT, TAPSE, and RVWT, whereas *Bmpr2*^+*/R899X*^ mice under normoxic conditions lacked these cardiovascular phenotypes (Fig. 5B–F). Preventive treatment with RAP-011 normalized each endpoint (Fig. 5B–F). In addition, exposure of *Bmpr2*^+*/R899X*^ mice to hypoxia for 5 weeks caused pulmonary macrophage infiltration (Fig. 5G,H), as determined by immunostaining for the macrophage marker F4/80. Similar to its therapeutic effects in the SuHxNx rat model, RAP-011 prevented infiltration of macrophages into the lungs of *Bmpr2*^+*/R899X*^ mice (Fig. 5G,H). However, in contrast to results in SuHxNx rats (Fig. 2A), we did not find evidence that molecular markers of inflammation are upregulated in the lungs of *Bmpr2*^+*/R899X*^ mice subjected to hypoxia (Supplemental Fig. 8), suggesting that the inflammatory phenotype in the mouse model is less severe. Together, these results indicate that a mouse model of heritable PAH, like the SuHxNx rat model of induced PAH described above, is characterized by marked inflammatory infiltrates, and that treatment with ActRIIA-Fc in either case is associated with suppressed macrophage infiltration and restored cardiopulmonary structure and function.

### Persistence of ActRIIA-Fc–induced cardiopulmonary benefits in severe experimental PAH

**Figure 6:**
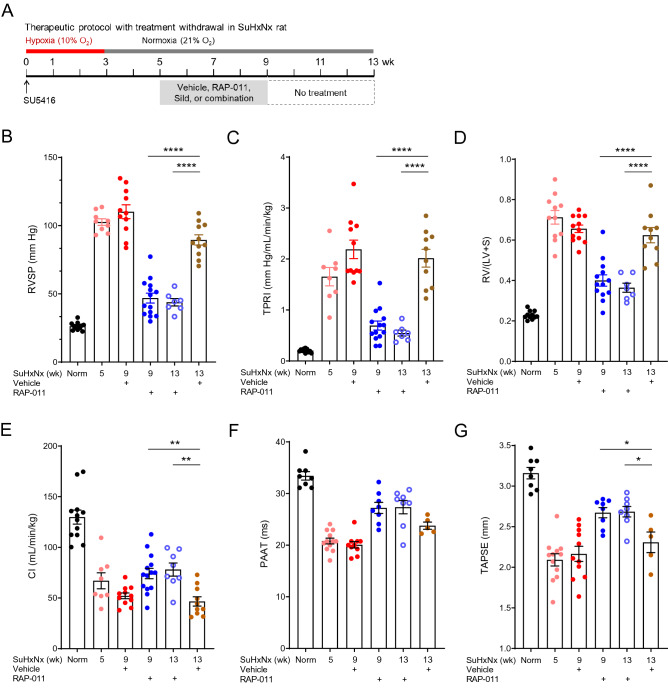
Disease-reversing effects of ActRIIA-Fc in severe experimental PAH persist after treatment cessation. (**A**) Experimental approach used to evaluate the persistence of therapeutic effects of RAP-011 in a SuHxNx rat model of severe PAH. Rats were treated on day 0 with a single dose of SU5416 (20 mg/kg, s.c.) and exposed to 3 weeks of normobaric hypoxia (10% O_2_) followed by 10 weeks of normoxia to allow disease progression. Rats were additionally treated twice weekly with RAP-011 (2.5 mg/kg, s.c.) or vehicle (PBS) from week 5 to week 9 post SU5416, at which time treatment was discontinued for the remaining 4 weeks. (**B**) RVSP, (**C**) TPRI, (**D**) Fulton index, (**E**) cardiac index (CI), (**F**) PAAT, and (**G**) TAPSE. Data are means ± SEM (n = 7–13 rats per group). Analysis by one-way ANOVA and Tukey post hoc test (*P < 0.05, **P < 0.01, ****P < 0.0001).

We investigated whether cardiopulmonary benefits of therapeutic RAP-011 treatment in severe experimental PAH are sustained after treatment cessation (Fig. 6A). In untreated SuHxNx rats, we confirmed that structural and functional abnormalities present by week 5, including altered RVSP, TPRI, RV hypertrophy, cardiac index, PAAT, and TAPSE, remain largely unchanged at week 13 (Fig. 6B–G). Therapeutic treatment with RAP-011, starting at week 5, produced significant improvement in these parameters by week 9 (Fig. 6B–G). Importantly, in SuHxNx rats treated therapeutically with RAP-011 from weeks 5 to 9, improvements in each of these endpoints persisted for 4 weeks after treatment cessation, until week 13 (Fig. 6B–G). Circulating levels of RAP-011 were undetectable 2 weeks after treatment was withdrawn. These results indicate that concurrent inhibition of activin-class ligands and blockade of inflammatory processes by ActRIIA-Fc leads to persistent reversal of cardiopulmonary structural remodeling in severe experimental PAH, even in the apparent absence of actively circulating therapeutic molecules.

## Discussion

The complex, multifactorial etiology of PAH represents a daunting challenge for development of disease-modifying therapies. Low penetrance of mutant alleles such as *BMPR2*^3,4^ indicates that factors in addition to BMPRII deficiency are required to induce PAH in most cases and underscores the importance of therapeutic approaches targeting multiple disease mediators in combination. We previously identified an unexpectedly prominent role of activin-class ligands as drivers of pulmonary vascular disease and established ActRIIA-Fc as a potential therapeutic approach for restoring the balance between pulmonary vascular SMAD1/5/8 and SMAD2/3 signaling in PAH^28^. Here, we identify activin-class ligands as key mediators of inflammatory and immune responses—either directly or indirectly—in severe experimental PAH and point to important regulatory effects of these ligands on macrophage activation and perivascular infiltration in diseased lung tissue. These results are the first to implicate activin-driven inflammation in pulmonary vascular remodeling in PAH and broaden the spectrum of known pathologic effects for these important TGF-β superfamily ligands.

Our results demonstrate that inflammatory gene signatures and macrophage perivascular infiltrates in severe experimental PAH were normalized by therapeutic treatment with ActRIIA-Fc, and the concordance we observed between aberrant gene profiles in this rodent model and in PAH patients strongly supports the translatability of these findings to human PAH. As confirmed by principal component analysis, therapeutic treatment with ActRIIA-Fc reversed this inflammatory pathologic phenotype to an extent far exceeding that of a standard vasodilator. ActRIIA-Fc similarly prevented macrophage infiltration in lung tissue while exerting beneficial cardiopulmonary effects in two other models, most notably a mouse model of heritable PAH arising from *Bmpr2* haploinsufficiency. Additionally, our results support a disease mechanism in which activins and GDFs with overlapping activity profiles act in a concerted manner to promote pulmonary inflammation and cardiopulmonary remodeling. Thus, whereas blockade of either activins or GDFs with their respective antibodies elicited structural and functional improvements, their combined neutralization conferred additive benefit. Finally, anti-remodeling effects of ActRIIA-Fc treatment in severe experimental PAH were undiminished by concurrent vasodilator therapy, indicating the potential of this agent as an effective add-on therapy as well as monotherapy. Beneficial effects were sustained for at least one month after treatment cessation, suggesting that ActRIIA-Fc, unlike standard vasodilators, could be disease modifying.

There is a growing consensus that early and persistent inflammation and altered immune responses underlie PAH pathophysiology. It has been proposed that advanced vascular remodeling might be reversible by approaches that address specific inflammatory and immune processes^2^. Consistent with the normal anti-inflammatory role of BMPRII in pulmonary endothelial cells^11–13^, inflammation has been implicated as a likely second hit required to induce severe vascular pathology in the context of reduced BMPRII signaling^14^. In one study, Tian and co-workers found that an acute inflammatory insult caused mesenchymal transdifferentiation by pulmonary endothelial cells (EndMT) through activation of canonical SMAD2/3 signaling, an effect which was reversible in vitro by knockdown of *Tgfbr1* (ALK5) or *Smad3* and reversible in *Bmpr2* mutant rats by a small-molecule inhibitor of ALK5. Their findings further implicate SMAD1/5/8- and SMAD2/3-pathway interactions as an important point for convergence of early pathogenic factors in PAH^10^; however, it is noteworthy that interventions targeting ALK5 or SMAD3 could also potentially inhibit signaling by activins, GDF8, and GDF11, which share downstream effectors with TGF-β.

Our results identify perivascular macrophages as an important cell type by which ActRIIA-Fc reverses pulmonary vascular remodeling in severe experimental PAH. Although their roles require further investigation, monocytes-macrophages are heavily implicated in PAH^45–49^, potentially orchestrating both the initiation and resolution of pulmonary inflammation. Activin A in particular is involved broadly in macrophage activation, inflammation, and fibrosis^33,50–52^. In PAH patients, alveolar macrophages produce activin A, and elevated levels of circulating activin A are predictive of patient mortality^25^. Our results indicate that activin-class ligands can exert distinct but overlapping effects on monocytes-macrophages, with one shared effect of activin A and GDF11 being increased expression of *Ccl2*, which encodes an important chemokine that promotes monocyte-macrophage chemotaxis and endothelial permeability^53^. In contrast with the CCL2-promoting effect of SMAD2/3 pathway activators, BMP9 and BMP10 inhibit release of CCL2 by pulmonary endothelial cells to promote vascular quiescence^54^. Reciprocal regulation of CCL2 by activin-class ligands and BMPs suggests that CCL2 could be a key mediator through which activin-class ligands disrupt vascular quiescence and promote pathologic remodeling—an elegant example of bidirectional homeostatic regulation by SMAD2/3 and SMAD1/5/8 pathways. In addition, the joint regulation of CCL2 by both activin A and GDF11 underscores the need to target multiple ligands concurrently to achieve a robust therapeutic outcome.

One prominent proinflammatory cytokine identified in our study is IL-6, whose gene expression is elevated in severe experimental PAH and normalized by ActRIIA-Fc therapy. In addition, we found that activin A, activin B, and GDF11 each increase *Il6* expression levels as one component of their proinflammatory effects on monocytes-macrophages in vitro. IL-6 mediates pulmonary macrophage activation by adventitial fibroblasts, is implicated in human PAH, and when overexpressed causes spontaneous development of PH in mice^47,55–59^. Furthermore, BMPRII- and IL-6–associated pathways display reciprocal regulation in pulmonary smooth muscle cells^60^, providing yet another possible link between BMPRII signaling and inflammation in PAH pathogenesis. The extensive evidence for a pathogenic role of IL-6 suggested that this cytokine could be targeted to reduce inflammation and thereby attenuate other PAH disease components. However, a clinical trial in patients with PAH failed to demonstrate robust hemodynamic benefits of tocilizumab, a monoclonal antibody directed against the IL-6 receptor^61^. Although follow-up study will be required in larger patient populations, these results suggest that targeting inflammation alone might be insufficient for treatment of PAH and underscore the complex, multifactorial mechanisms of PAH disease progression.

It is particularly noteworthy that ActRIIA-Fc treatment in severe experimental PAH reverses elevated pulmonary expression of *Grem1*, which encodes an endogenous BMP antagonist (gremlin-1) regarded as an important promoter of vascular remodeling in PAH^38^. Hypoxia stimulates gremlin secretion by pulmonary microvascular endothelial cells, and *Grem1* haploinsufficiency reduces pulmonary vascular remodeling in mice exposed to chronic hypoxia^38^. Other cell types, including arterial smooth muscle cells and macrophages, could also be sources of gremlin-1, and the former type exhibits increased gremlin-1 expression in response to mechanical stretch in vitro^62,63^. Best known for its pro-proliferative actions, gremlin-1 has also been linked to inflammation in the kidney and the lung through effects on Notch signaling and macrophage migration, respectively^62,64^. Gremlin-1 was found to play vital roles in PAH associated with congenital heart disease (systemic-to-pulmonary shunts), which does not typically arise from *BMPR2* mutation; in this case, gremlin-1 could help to explain reduced BMPRII–SMAD1/5/8 pathway activity in the presence of intact *BMPR2*^63^. Importantly, therapeutic immunoneutralization of gremlin-1 reduces pulmonary vascular remodeling in experimental PAH^65^. Thus, reversal of *Grem1* overexpression could be a key mechanism by which ActRIIA-Fc rebalances SMAD1/5/8 signaling with SMAD2/3 signaling in the pulmonary vasculature^28^. Because gremlin-1 acts as a mediator of BMPRII pathway inhibition by endothelin^66^, gremlin-1 modulation could also be a potential point of mechanistic convergence between therapeutic effects of ActRIIA-Fc and those of endothelin receptor antagonists in patients with PAH.

Vascular remodeling in PAH is understood in broad terms to arise from physiologic cellular responses to stress or injury that eventually become dysregulated and persistent. Inflammation, hypoxia, or biomechanical stress in individuals with impaired BMPRII pathway activity may promote pathologic remodeling of the extracellular matrix, abnormal cellular proliferation, and potentially EndMT^67^. Recent findings indicate that BMPRII plays a protective role in endothelial cell homeostasis, with loss of BMPRII favoring EndMT and driving cells toward a primed biomechanical state in which changes in stiffness or shear stress provide a second hit and initiate a self-sustaining cycle of excess TGF-β signaling^10^. Chronic TGF-β1 signaling in PAH also induces sustained SMAD3 activation in pulmonary artery smooth muscle cells, which correlates with the hemodynamic and morphologic PAH phenotype in rodents^68^. Extensive involvement of TGF-β in these important disease processes raises the possibility that other SMAD2/3-pathway ligands may play under-appreciated pathologic roles that reinforce or overlap with those of TGF-β. Indeed, recent findings indicate that activin A produced by pulmonary microvascular endothelial cells can promote PAH through increased internalization and degradation of BMPRII^29^, in yet another example of crosstalk between SMAD2/3 and SMAD1/5/8 pathway branches.

In this report, we compared the effects of ActRIIA-Fc with those of the phosphodiesterase type 5 inhibitor sildenafil in PAH models and found far greater anti-inflammatory efficacy for ActRIIA-Fc. However, vasodilator therapies approved for use in PAH can act through one of several distinct mechanisms, also including stimulation of soluble guanylate cyclase and inhibition of endothelin receptors. Our present results should therefore be interpreted conservatively, as further studies will be required to compare anti-inflammatory effects of ActRIIA-Fc with those of other vasodilator classes, both individually and in combination. In addition, clinical data will be required to determine whether the anti-inflammatory effects seen with ActRIIA-Fc treatment in rodent models also contribute to efficacy in patients with PAH.

Complete reversal of PAH in rare cases^69^ offers hope that cardiopulmonary remodeling in PAH patients can be reversed more generally with a sufficiently robust therapeutic approach. The ability of ActRIIA-Fc to reverse established pulmonary inflammation and cardiopulmonary remodeling in severe experimental PAH indicates that the SMAD2/3-pathway ligands targeted by this agent mediate key interactions between cell types implicated in this disease, potentially including perivascular immune cells, endothelial cells, vascular smooth muscle cells, and adventitial fibroblasts. We speculate that the breadth and robustness of ActRIIA-Fc activity revealed here in experimental PAH, attained through mutually reinforcing effects on pathogenic components of inflammation and cardiopulmonary remodeling, could potentially translate to disease-modifying activity of sotatercept in patients with PAH, as either a monotherapy or add-on to currently available therapies for PAH.

## Materials and methods

All experiments were performed in accordance with the relevant guidelines and regulations approved by Acceleron Pharma Inc., a subsidiary of Merck & Co., Inc., Kenilworth, NJ, USA.

### Animal studies

Animal studies were approved by the Institutional Animal Care and Use Committee at Acceleron Pharma Inc., a subsidiary of Merck & Co., Inc., Kenilworth, NJ, USA, in accordance with ARRIVE guidelines. Adult male Sprague–Dawley (SD) and Wistar (WI) rats (150–180 gm) (Envigo, Indianapolis, IN) were used as SuHxNx and MCT rat models, respectively. The SuHxNx model was established by a single subcutaneous injection of vascular endothelial growth factor receptor antagonist semaxanib (SU5416, 20 mg/kg; Cayman Chemical) with immediate onset of exposure to normobaric hypoxia (10% O_2_) for 3 weeks followed by normoxia (21% O_2_) for 6 weeks as described^34,35,70^. Sildenafil was obtained from Cayman Chemical. The MCT model was established by a single subcutaneous injection of MCT (60 mg/kg, Torris) followed by 4 week exposure to normoxia. *Bmpr2*^+*/R899X*^ mice were generated as a model of BMPR2 haploinsufficiency as described^44^.

### Fusion protein and neutralizing antibodies

RAP-011 was constructed and purified essentially as described^71^. Anti-activin A antibody (A2)^72^ and an antibody with dual specificity for myostatin and GDF11 (RK35)^73^ were modified internally for use in mice by substitution of murine IgG2a Fc. Anti-activin B antibody was generated using the Adimab platform and validated internally.

### Hemodynamic and RV measurements

Animals were anesthetized with 3–4% isoflurane and placed on controlled heating pads. RV systolic pressure (RVSP) was measured by advancing a curved-tip pressure transducer catheter, 2F (SPR-513, Millar Instruments) for rats and 1F (SPR-1000, Millar Instruments) for mice, into the RV via the right jugular vein under 1–2% isofluorane anesthesia. In rats, cardiac output was assessed by advancing a 2F microtipped PV catheter (SPR 838, Millar Instruments) into the left ventricle through the right carotid artery under 1.5–2% isofluorane anesthesia. Cardiac index (CI) was calculated by dividing cardiac output by body weight. Total pulmonary vascular resistance index (TPRI) was estimated by dividing RVSP by CI^35^. Heart and lungs were collected en bloc and lungs were perfused with physiological saline via the RV outflow tract to flush the blood cells from the pulmonary circulation. RV hypertrophy was determined by calculating the weight ratio of the RV free wall to the combined left ventricle and septum (Fulton index). Values shown for hemodynamic parameters and Fulton index from rats treated with sildenafil monotherapy are historical data from our laboratory.

### Histopathology and immunohistochemistry

After perfusion, the right pulmonary lobe was separated and snap frozen for biochemical analysis while the left pulmonary lobe was collected and preserved in neutral buffered formalin as described^34,35^. The left lobe was blocked and embedded in paraffin. Sections of formalin-fixed paraffin-embedded (FFPE) lungs were treated with hematoxylin and eosin (H&E), Verhoeff-Van Gieson stain, or Masson’s trichome stain for histological analysis. Immunohistochemical staining was performed using antibodies against αSMA (19245, Cell Signaling Technology), CD11b (ab133357, Abcam), and F4/80 (70076, Cell Signaling Technology). Numbers of CD11b-positive and F4/80-positive cells were counted in a blinded manner in 30–40 randomly selected fields at high magnification with HALO image analysis software and expressed as a percentage of total cells.

### Immunoblotting

Frozen tissue samples were pulverized and homogenized in RIPA buffer (Sigma, cat# R0278) with short pulses for 3 min at 4 °C. Homogenates were kept on ice for 20 min, and protein quantification was performed with BCA assay (Thermo Fisher, cat# A53225). 20–30 µg of protein was used for gel electrophoresis on a 4–15% gel (Bio-Rad, cat# 4,568,085) and transferred to nitrocellulose membranes at 250 mA for 90 min. The membranes were incubated overnight at 4 °C with antibodies against BMPRII (Invitrogen, cat# MA5-15,827), pSmad1/5 (Invitrogen, cat# 700,047), pSmad2/3 (Cell Signaling, cat# 8828), and GAPDH (Cell Signaling, cat# 5174). Signal was detected using HRP-conjugated secondary antibodies (Invitrogen). Densitometry was performed using ImageJ software. Uncropped immunoblot gel images are presented in Supplemental Fig. 9 and Supplemental Fig. 10.

### Morphological analyses

Lung sections prepared with modified Verhoeff-Van Gieson stain and anti-αSMA immunostaining were used to assign grades for vascular occlusion and analyzed with HALO software (Indica Labs) to determine wall thickness of pulmonary arteries. Wall thickness was expressed as [(OD – ID)/OD] × 100%, where OD is vessel outer diameter and ID is inner diameter. Briefly, each lung section was subdivided by rectangular grid, and outer and inner perimeters of vessels in randomly selected rectangles were measured to calculate OD and ID, respectively. Wall thickness was stratified on the basis of OD into three groups, < 50 μm, 50–100 μm, and > 100 μm. The degree of vascular occlusion was classified as grade 0 (no luminal occlusion), grade 1 (< 50% luminal occlusion), or grade 2 (> 50% luminal occlusion) as described^35^. Approximately 100 arteries per lung section were analyzed in each of four animals per treatment group. All measurements and scoring of occlusive lesions were performed by an investigator blinded to treatment grouping.

### Echocardiography

Echocardiography was performed with a Vevo 3100 imaging system with MX201 scanhead (VisualSonics, Toronto, ON, Canada) on rats anesthetized with 3–4% isoflurane and maintained with 1.5–2% isoflurane. B-Mode, M-Mode and pulse-wave Doppler flow imaging were performed in each rat at the end of weeks 5 and 9. Briefly, rats were placed supine on a heated platform and allowed to breathe spontaneously. The RV outflow tract was visualized using a modified parasternal long axis view. Pulmonary artery acceleration time (PAAT) was measured as the time from start to peak velocity of blood flow in the lumen of the main pulmonary artery distal to the pulmonary valve as obtained from the pulse-wave doppler recording. B-Mode parasternal short-axis view of a mid-ventricular cross section of the heart was visualized at the level of the papillary muscles. RV wall thickness (RVWT) was measured using M-mode in a modified parasternal long-axis view through the aortic valve. RV fractional area change (RVFAC) was measured using a B-mode apical four-chamber view. Tricuspid annular plane systolic excursion (TAPSE) was obtained from the apical four-chamber view directing the M-mode doppler beam through the lateral annulus of the tricuspid valve plane. For each parameter, measurements from three individual heartbeats per animal were taken and averaged. Values shown for echocardiographic parameters PAAT, RVWT, and TAPSE from rats treated with sildenafil monotherapy are historical data from our laboratory.

### Quantitative PCR

Frozen tissues were pulverized using a stainless steel mortar and pestle (Cellcrusher) chilled in liquid nitrogen and homogenized in 700 μl QIAzol (79306, QIAGEN) with a Precellys CK28-R hard tissue homogenizing kit (P000916-LYSK0A, Bertin). Total RNA was extracted from homogenized tissues by using the miRNeasy Mini Kit (217994, QIAGEN), and the concentration and quality of RNA was determined by absorbance at 260/280 nm with a NanoDrop One Spectrophotometer (Thermo Scientific, USA). cDNA was prepared using RNA to cDNA EcoDry™ Premix-Random Hexamers (639546, Takara Bio, USA). Quantitative PCR was performed on reverse-transcribed cDNA using TaqMan™ Universal PCR Master mix (4304437, Applied Biosystem) to analyze mRNA expression levels. Relative expression of mRNA was determined by the ΔΔCt method.

### RNA sequencing

Total lung RNA was isolated from frozen tissue with a miRNeasy mini kit (Qiagen). RNA samples of 100 ng were used to prepare amplified cDNA libraries using the Universal Plus mRNA-Seq Library Prep Kit (NuGEN Technologies, Inc.). Libraries were then sequenced with 2 × 150 bp paired-end configuration on Illumina HiSeq 4000 with a single index read. RNA-seq datasets have been deposited in SRA (PRJNA637249).

### Cell culture

Human monocytic cells THP-1 (TIB-202, ATCC) were maintained in Roswell Park Memorial Institute medium (RPMI 1640) (30-2001, ATCC) supplemented with 2-mercaptoethanol (31,350,010, Gibco) to a final concentration of 0.05 mM, fetal bovine serum (30-2020, ATCC) to a final concentration of 10% and penicillin–streptomycin-amphotericin B solution (PCS-999–002, ATCC) at a dilution of 1:1000. To assess macrophage activation, 1.5 × 10^6^ THP-1 cells were treated with activn A (5 ng/mL) or GDF11 (5 ng/mL) for 24 h, or activin B (50 ng/mL) for 6 h, in serum-free media. Prior to treatment, cells were growth-arrested overnight in serum free media. After treatment, cells were washed twice with ice-cold PBS and lysed with Buffer RLT plus (1,053,393, Qiagen). Total RNA was extracted with RNAeasy plus kit (74,134, Qiagen) according to the manufacturer’s instructions. cDNA was prepared using RNA-to-cDNA EcoDry™ Premix-Random Hexamers (639,546, Takara Bio, USA). Quantitative PCR was performed on reverse-transcribed cDNA using TaqMan™ Universal PCR Master mix (4,304,437, Applied Biosystem) to analyze mRNA expression levels. Relative expression of mRNA was determined by the ΔΔCt method.

### Pulmonary cell suspensions and fluorescence-activated cell sorting (FACS)

Single-cell suspensions were prepared from lung as described^74^. Briefly, heart and lungs were collected en bloc, and lungs were perfused with phosphate buffered saline (PBS) via the RV outflow tract to flush blood cells from the pulmonary circulation. After perfusion, lung lobes were diced for 2 min in 20 ml ice-cold digestion buffer (HBSS), containing 1.5 mg/ml collagenase A (Roche), 0.4 mg/ml DNase I (Roche), 5% fetal bovine serum, and 10 mM HEPES, pH 7.4. This tissue suspension was incubated at 37 °C for 30 min with continuous gentle shaking. After digestion, 20 ml of ice-cold PBS was added and gently mixed. The resulting cell suspension was strained through a 70 μm cell strainer, centrifuged at 12,000 rpm at 4 °C for 5 min, and treated with red blood cell lysis buffer on ice for 10 min. Following red blood cell lysis, remaining cells were washed twice with PBS at 12,000 rpm at 4 °C for 5 min. For FACS, cells were incubated in 100 µl of flow mix solution composed of stain buffer (BD Biosciences) with 2% Fc block containing antibodies at 1:33 fold dilution (except for stained controls) for 30 min at 4 °C. After staining, cells were washed with 1 ml of stain buffer twice at 12,000 rpm at 4 °C for 5 min. The cell pellet was resuspended in 500 µl stain buffer, and propidium iodide was added to allow exclusion of dead cells. Data was acquired with an LSRII flow cytometer (BD Biosciences) using FACSDiva software (BD Biosciences). Compensation was performed on the flow cytometer at the beginning of each experiment. Data were analyzed using Flowjo v10.

### Statistical analysis

Statistical analysis was performed using GraphPad Prism 8. Data are reported as means ± SEM. Differences between groups were analyzed using Student’s *t*-test or ANOVA with Tukey post hoc test for multiple comparisons. Differences were considered significant at *P* < 0.05.

## Supplementary Information

Supplementary Information.
