# Supplementary material for: Sotatercept analog suppresses inflammation to reverse experimental pulmonary arterial hypertension
Source: Sci Rep. 2022 May 12;12:7803. doi: 10.1038/s41598-022-11435-x (PMC9098455; doi:10.1038/s41598-022-11435-x)
Supplement: Supplementary file 1 — Supplementary Information. [file 41598_2022_11435_MOESM1_ESM.pdf]

Sotatercept analog suppresses inflammation to reverse experimental pulmonary arterial hypertension

Sachindra R. Joshi[1], Jun Liu[1], Troy Bloom[1,3], Elif Karaca Atabay[1], Tzu-Hsing Kuo[1], Michael Lee[1], Elitza Belcheva[1], Matthew Spaits[1], Rosa Grenha[1], Michelle C. Maguire[1], Jeffrey L. Frost[1], Kathryn Wang[1], Steven D. Briscoe[1], Mark J. Alexander[1], Brantley R. Herrin[1], Roselyne Castonguay[1], R. Scott Pearsall[1,4], Patrick Andre[1], Paul B. Yu[2], Ravindra Kumar[1], Gang Li[1]*

[1] Discovery Group, Acceleron Pharma Inc., a subsidiary of Merck & Co., Inc., Kenilworth, NJ, USA.

[2] Division of Cardiovascular Medicine, Department of Medicine, Brigham and Women's Hospital, Harvard Medical School, Boston, MA 02115, USA.

[3] Present address: Ultivue, Cambridge, MA, USA

[4] Present address: Cellarity, Cambridge, MA, USA

* Correspondence:

garygl@gmail.com

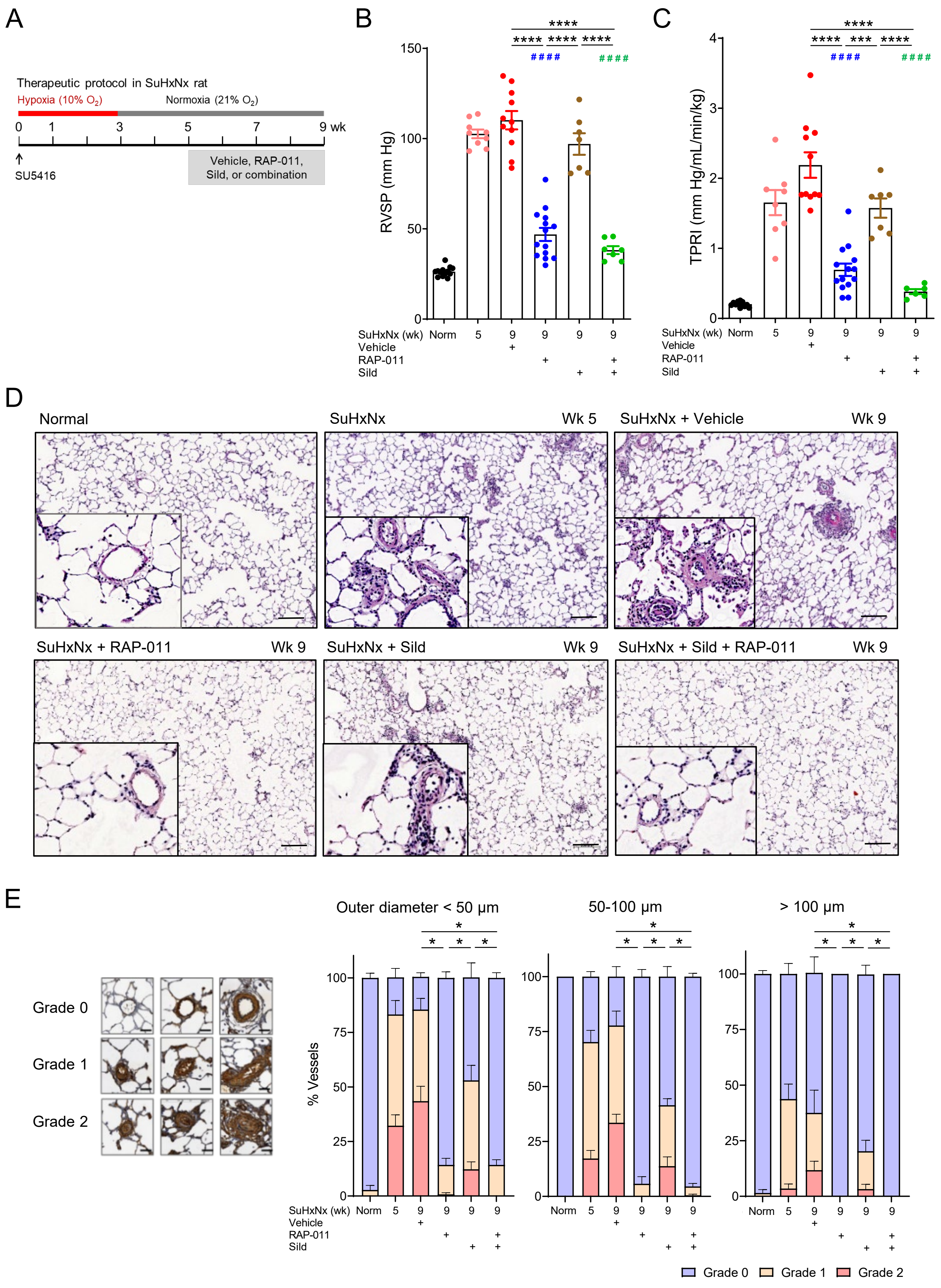

A
Therapeutic protocol in SuHxNx rat
Hypoxia (10% O2)
Normoxia (21% O2)
0 1 3 5 7 9 wk
SU5416
Vehicle, RAP-011, Sild, or combination
B
RVSP (mm Hg)
C
TPRI (mm Hg/mL/min/kg)
SuHxNx (wk) Norm 5 9 9 9 9
Vehicle
RAP-011
Sild
D
Normal
SuHxNx Wk 5
SuHxNx + Vehicle Wk 9
SuHxNx + RAP-011 Wk 9
SuHxNx + Sild Wk 9
SuHxNx + Sild + RAP-011 Wk 9
E
Grade 0
Grade 1
Grade 2
Outer diameter < 50 µm
50-100 µm
> 100 µm
% Vessels
SuHxNx (wk) Norm 5 9 9 9 9
Vehicle
RAP-011
Sild
Grade 0 Grade 1 Grade 2

Supplemental Figure 1. **ActRIIA-Fc is effective in combination therapy as well as monotherapy for reversing pulmonary vascular remodeling in severe experimental PAH.** (**A**) Experimental approach used to evaluate therapeutic effects of RAP-011 in a Sugen-hypoxia-normoxia (SuHxNx) rat model of severe PAH. Rats were treated on day 0 with a single dose of SU5416 (20 mg/kg) and exposed to normobaric hypoxia (10% O_2) for 3 weeks followed by 6 weeks of normoxia to allow disease progression. Rats were additionally treated with RAP-011 (2.5 mg/kg, s.c., twice weekly), sildenafil (30 mg/kg, p.o., twice daily), combination therapy with RAP-011 and sildenafil, or vehicle (PBS) for 4 weeks starting on week 5 post SU5416. (**B**) RVSP and (**C**) total pulmonary resistance index (TPRI). Data are means ± SEM (n = 7-14 per group) (***P < 0.001, ****P < 0.0001; $^{####}$ P <0.0001 vs. wk 5). (**D**) Images of representative lung sections stained with hematoxylin and eosin. Scale bar, 200 µm. (**E**) Images of lung sections immunostained with an antibody against α-smooth muscle actin to illustrate grades of pulmonary histopathology. Scale bar, 50 µm. Percentage of pulmonary arterial vessels classified as grade 0 (normal, no occlusion), grade 1 (< 50% occlusion), or grade 2 (> 50% occlusion) grouped according to vessel outer diameter. Data are means ± SEM (n = 4 rats per group). Analysis by one-way ANOVA and Tukey post hoc test; for clarity, only significance for percentage of grade 0 vessels is indicated (*P < 0.05).

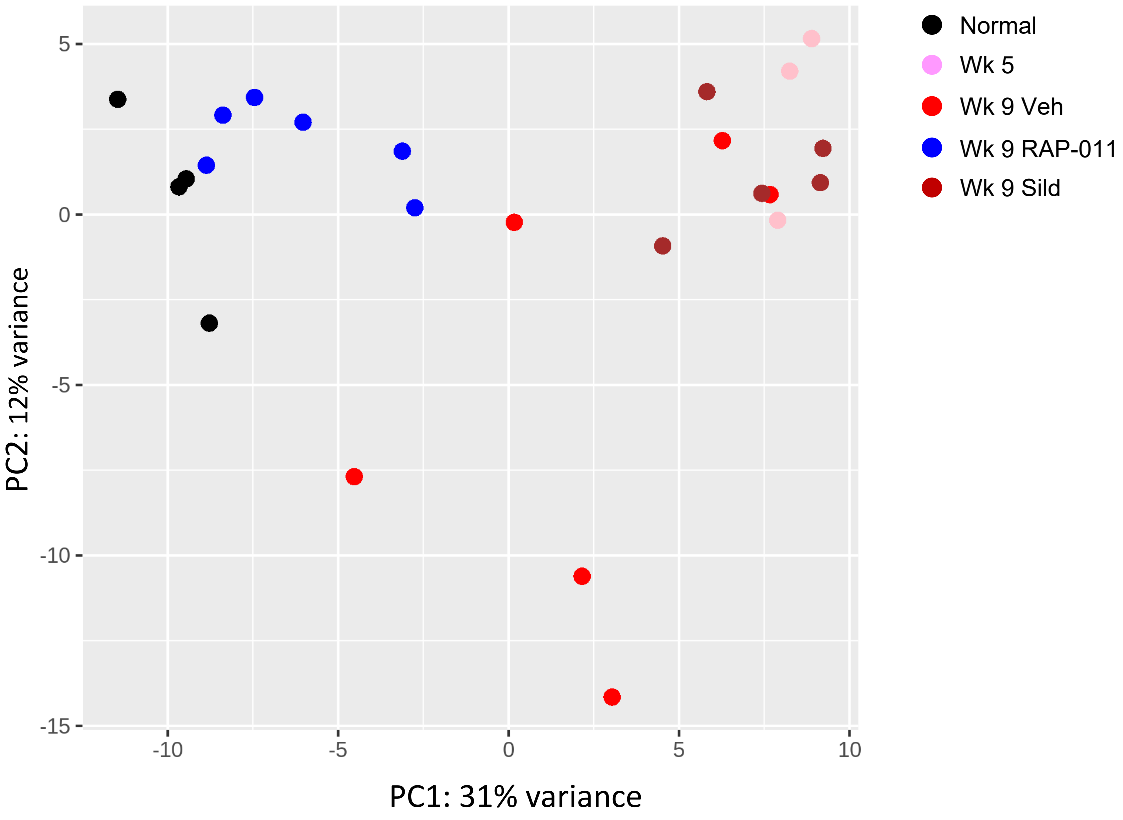

Supplemental Figure 2. Principal component analysis of DEGs in lung as a function of therapeutic treatment in a SuHxNx rat model of severe PAH. Symbols represent normal rats, untreated SuHxNx rats (Wk 5), and SuHxNx rats treated therapeutically with RAP-011 (Wk 9 RAP-011), sildenafil (Wk 9 Sild), or vehicle (Wk 9 Veh). Variance profile of DEGs based on the top two components indicates that RAP-011–treated rats most closely resemble normal rats, whereas sildenafil-treated rats resemble SuHxNx rats without therapeutic intervention.

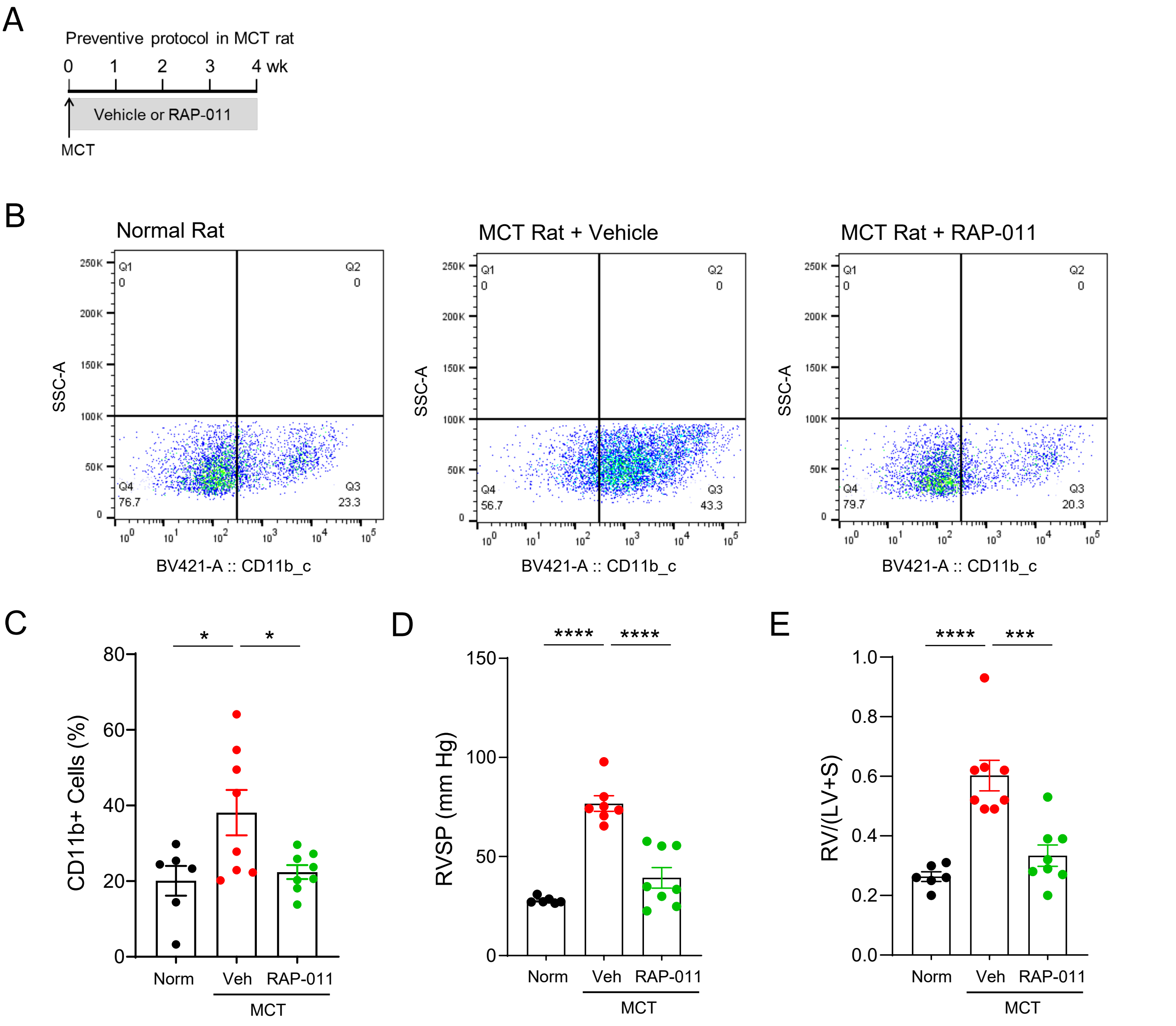

Supplemental Figure 3. **ActRIIA-Fc blocks pulmonary macrophage infiltration and improves cardiopulmonary function in a monocrotaline-treated rat model of PH.** (**A**) Experimental approach used to test effects of RAP-011 in a monocrotaline (MCT) rat model of PH. Rats were treated on day 0 with a single dose of MCT (60 mg/kg, s.c.) then treated twice weekly with RAP-011 (5 mg/kg, s.c.) or vehicle (PBS) for 4 weeks starting on day 0. (**B**) Representative flow-cytometry plots of pulmonary CD11b+ cells in normal or MCT rats. (**C**) Percentage of CD11b+ cells in lung determined by flow cytometry. (**D**) RV systolic pressure (RVSP) and (**E**) Fulton index, calculated as the ratio of RV weight to weight of the combined left ventricle and septum (LV+S), in MCT rats. Data are means ± SEM (n = 6–8 rats per group). Analysis by one-way ANOVA and Tukey post hoc test. *$P < 0.05$, ***$P < 0.001$, ****$P < 0.0001$.

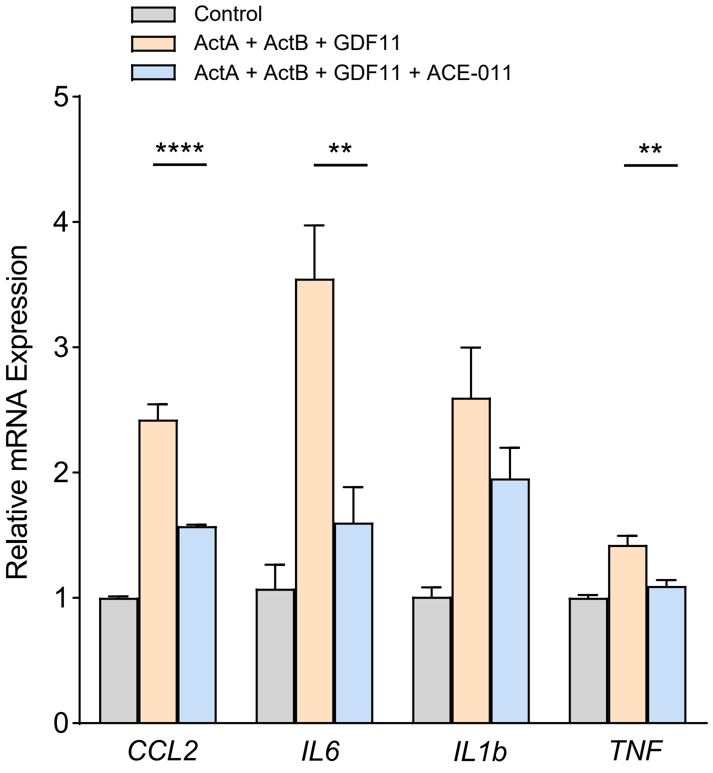

Supplemental Figure 4. ActRIIA-Fc inhibits cytokine expression induced by combined activin-class ligands in THP-1 cells *in vitro*. Effect of ACE-011 co-treatment on increased expression of *CCL2*, *IL6*, *IL1b*, and *TNF* induced by combined treatment with activin A (ActA), activin B (ActB), and GDF11 in THP-1 cells. Analysis by one-way ANOVA and Dunnett post hoc test; $^{**}P < 0.01$, $^{****}P < 0.0001$.

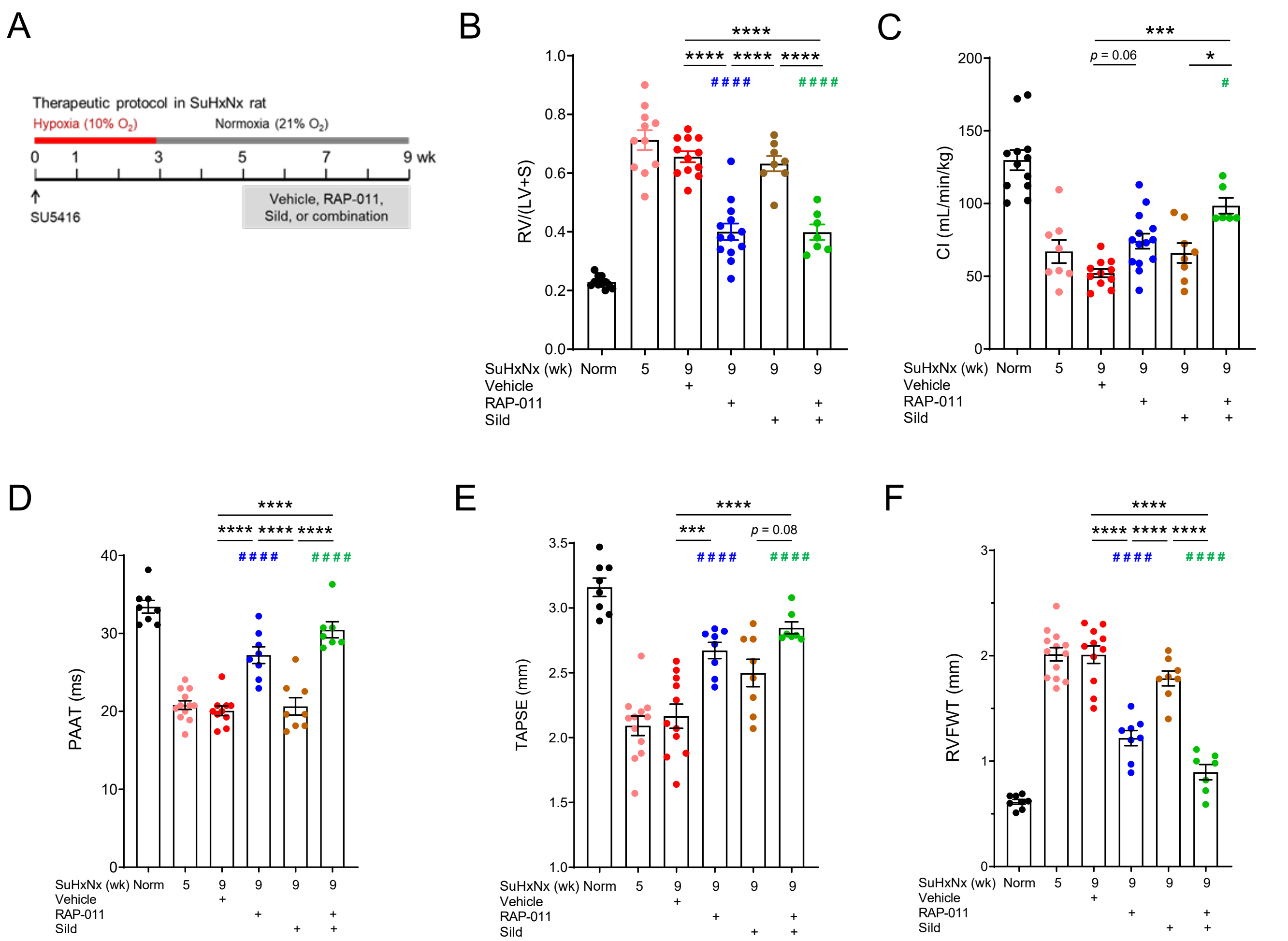

Supplemental Figure 5. ActRIIA-Fc is effective in combination therapy as well as monotherapy for reversing cardiac remodeling in severe experimental PAH. (**A**) Experimental approach used to evaluate therapeutic effects of RAP-011 in a SuHxNx rat model of severe PAH. See Supplemental Figure 1 for details. (**B**) Fulton index, calculated as the ratio of RV weight to weight of the combined left ventricle and septum (LV+S), and (**C**) cardiac index (CI) in normal or SuHxNx rats. Data are means ± SEM (n = 7-13 rats per group). (**D**) Pulmonary artery acceleration time (PAAT), (**E**) tricuspid annular plane systolic excursion (TAPSE), and (**F**) RV free-wall thickness (RVFWT) measured at diastole. Data are means ± SEM (n = 7-11 rats per group). Analysis by one-way ANOVA and Tukey post hoc test (*$P < 0.05$, **$P < 0.01$, ***$P < 0.001$, ****$P < 0.0001$; ### $P < 0.001$ vs. wk 5, #### $P < 0.0001$ vs. wk 5).

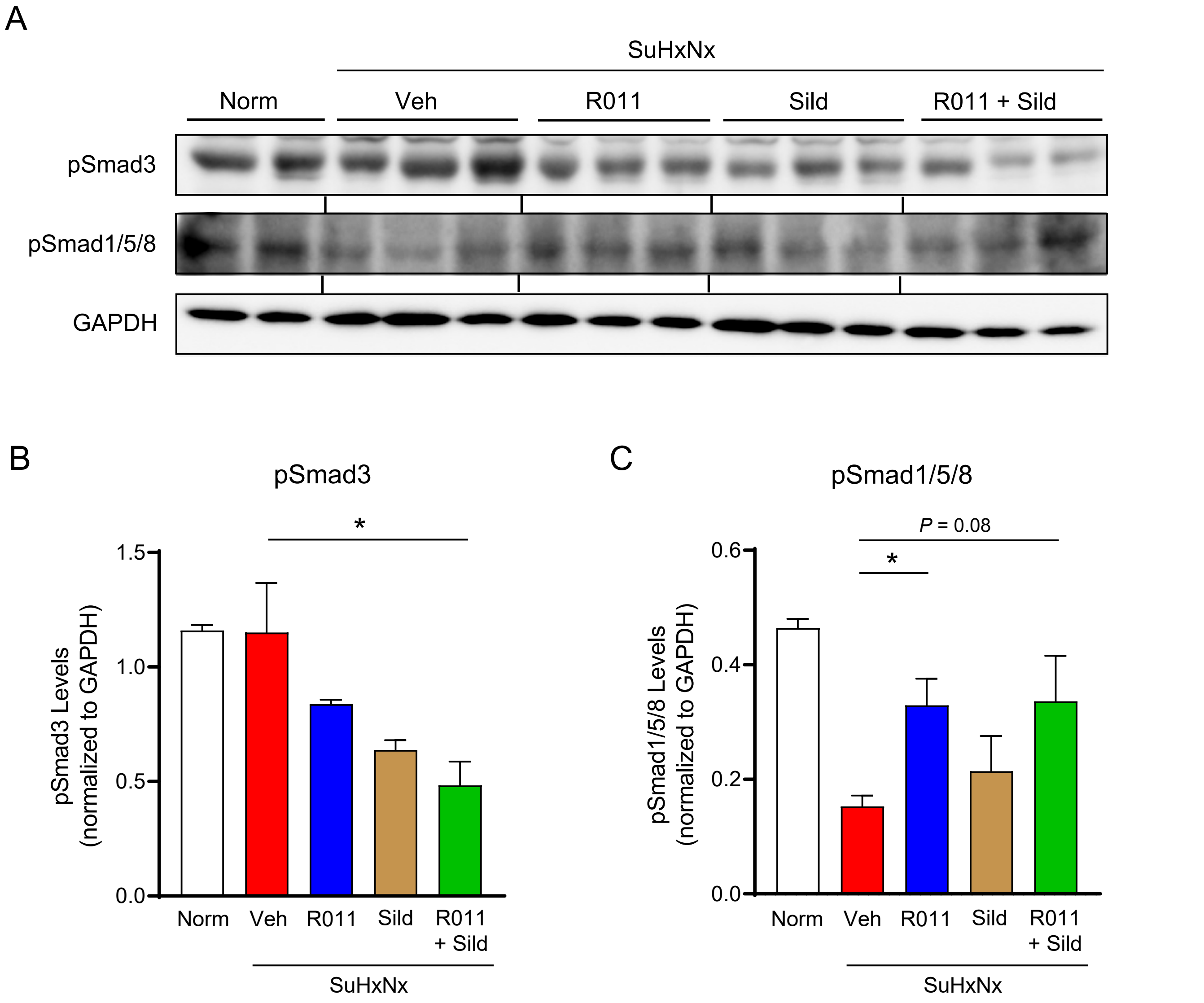

Supplemental Figure 6. ActRIIA-Fc monotherapy and combination therapy with a vasodilator correct Smad pathway activation imbalance in the RV in severe experimental PAH. (**A**) Levels of pSmad3 and pSmad1/5/8 protein in the RV of SuHxNx rats treated with RAP-011 (R011), sildenafil (Sild), RAP-011 and sildenafil in combination, or vehicle (Veh) as in Supplemental Figure 1A. Norm, normal controls. (**B**) Quantification of normalized pSmad3 levels. (**C**) Quantification of normalized pSmad1/5/8 levels. Analysis by one-way ANOVA and Dunnett post hoc test; * $P < 0.05$.

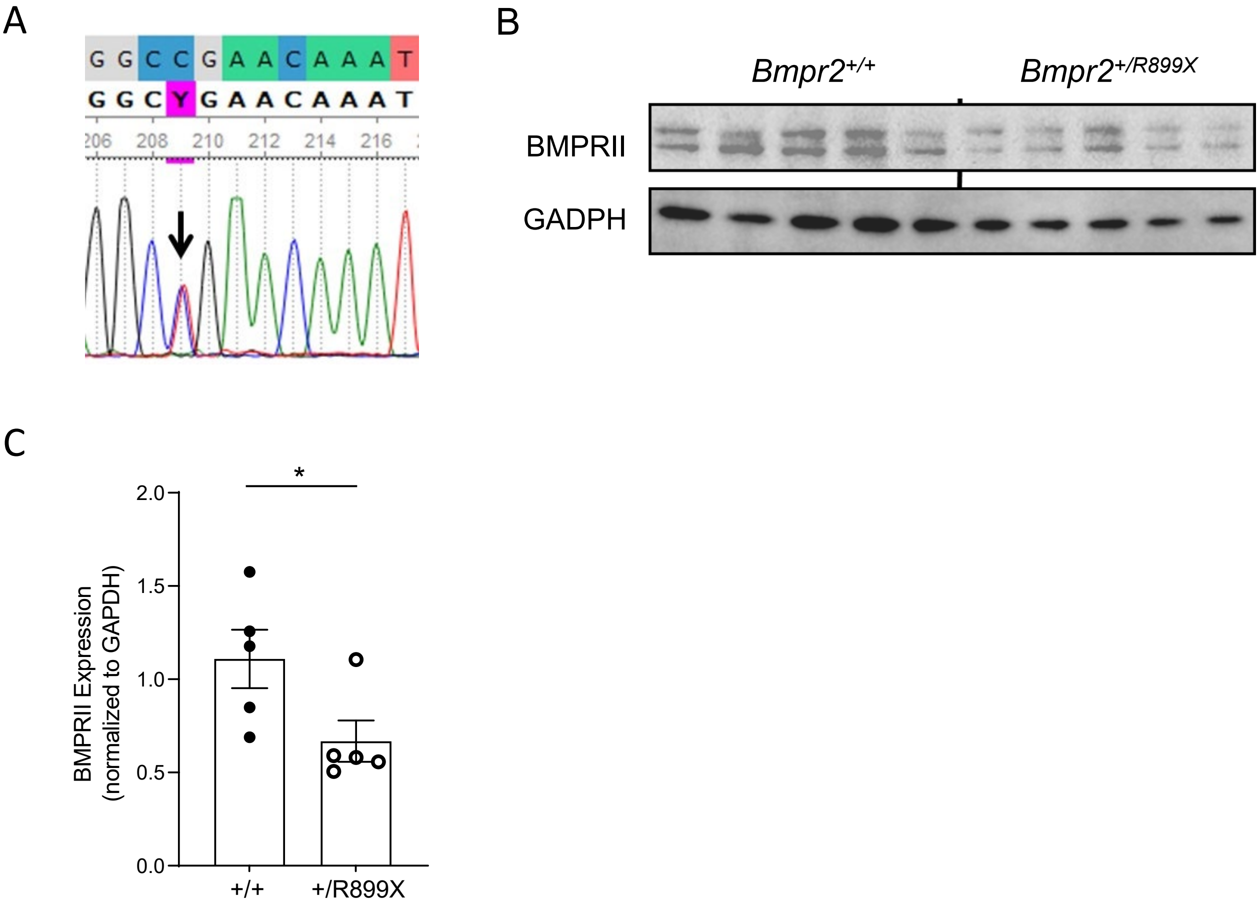

Supplemental Figure 7. Confirmation of *Bmpr2* haploinsufficiency in *Bmpr2*$^{+/R899X}$ mice. (**A**) Amplification of genomic DNA by PCR and direct sequencing confirmed the presence of a heterozygous mutation (arrow) in *Bmpr2*$^{+/R899X}$ mice (equal peak heights for wild-type and mutant alleles). (**B**) Immunoblot of lung homogenates from wild-type and *Bmpr2*$^{+/R899X}$ mice analyzed to determine expression of BMPRII (detected by MA5-15827, Invitrogen). (**C**) Quantification of BMPRII protein normalized to GADPH. Data are means ± SEM (n = 5 per group). Analysis by Students t-test. *P < 0.05.

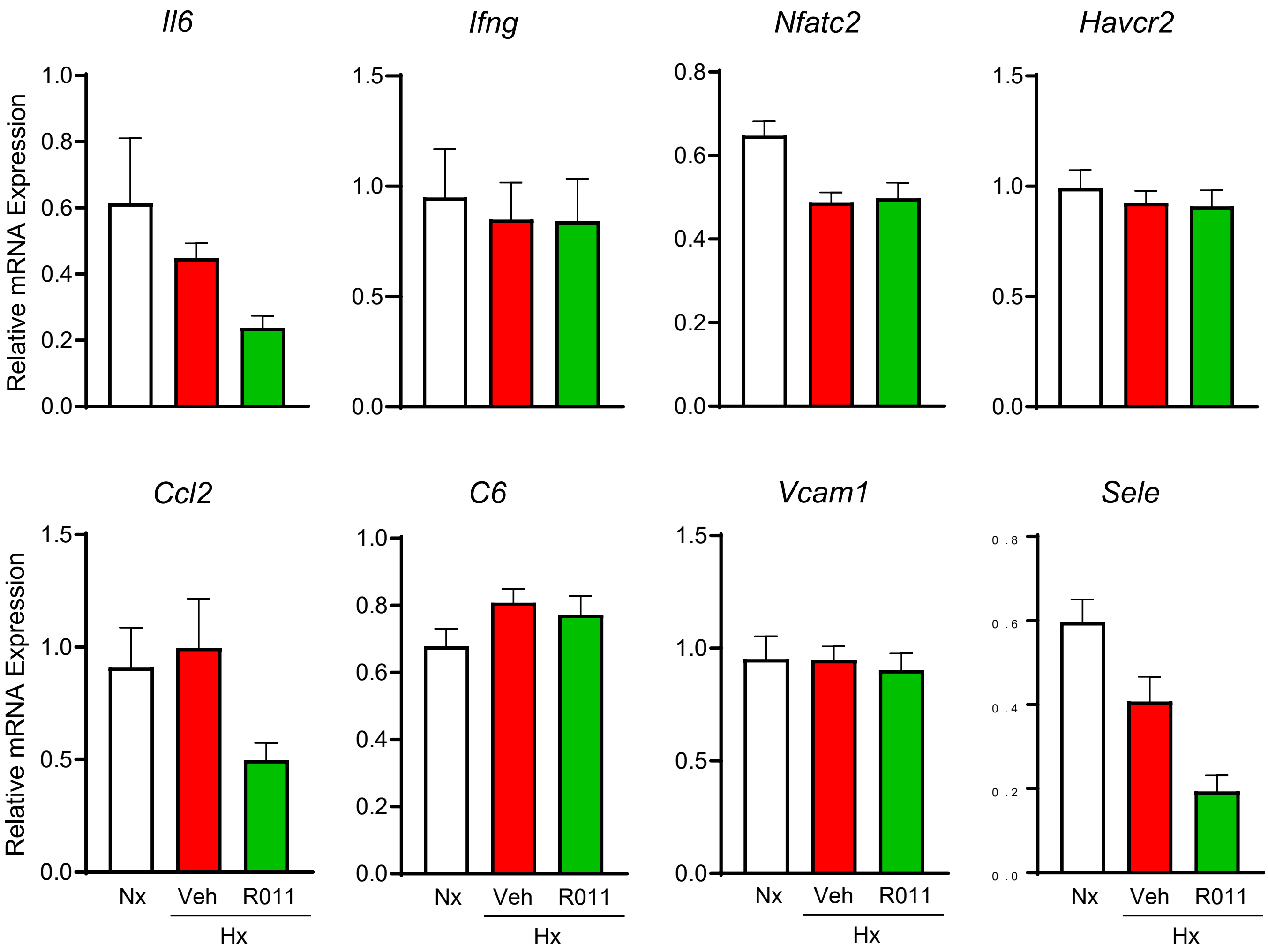

Supplemental Figure 8. Mouse model of *Bmpr2* hapoloinsufficiency with hypoxia does not display upregulation of selected inflammatory markers in lung. Effects of RAP-011 (R011) on cytokine mRNA expression in *Bmpr2*$^{+/R899X}$ mice under hypoxic conditions (Hx) as indicated in Figure 5A. Nx, normoxic controls; Veh, vehicle.

A

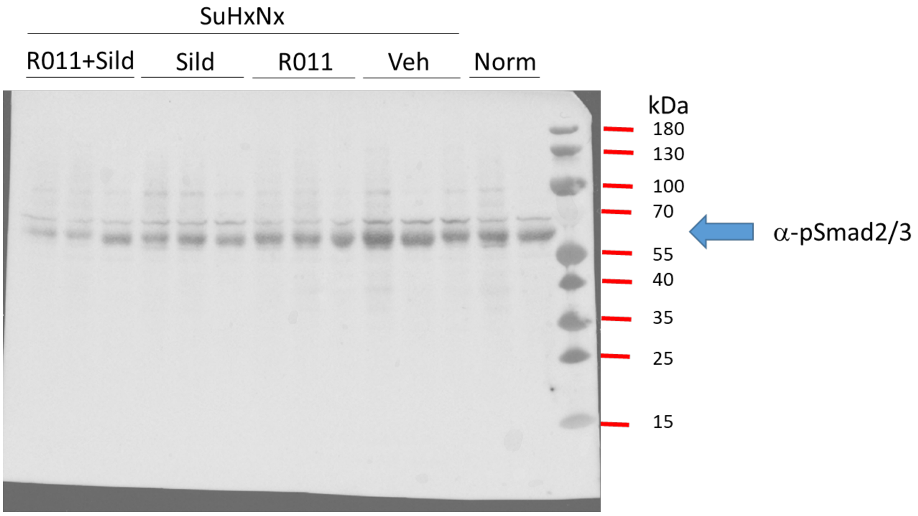

B

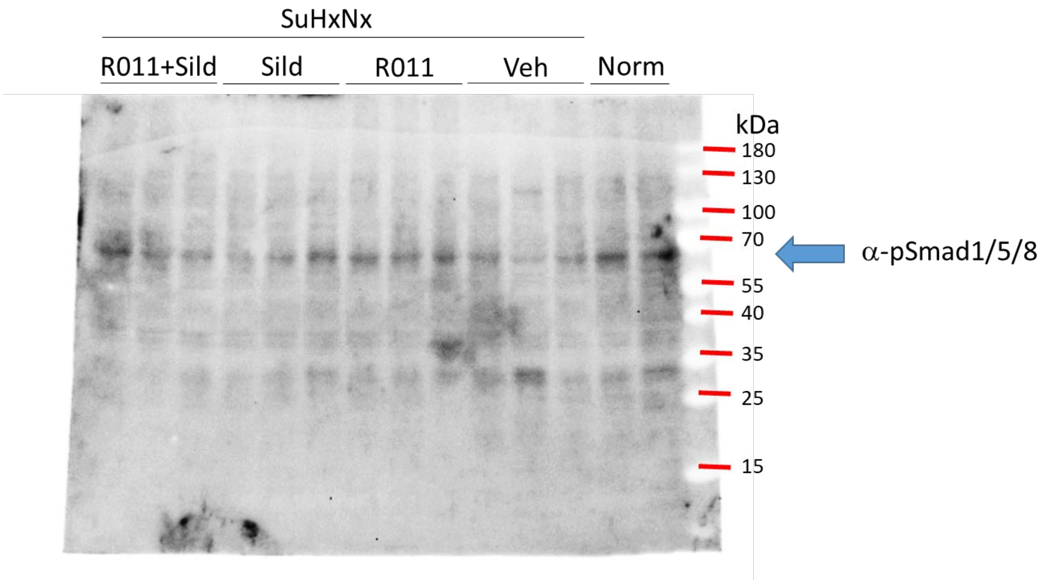

C

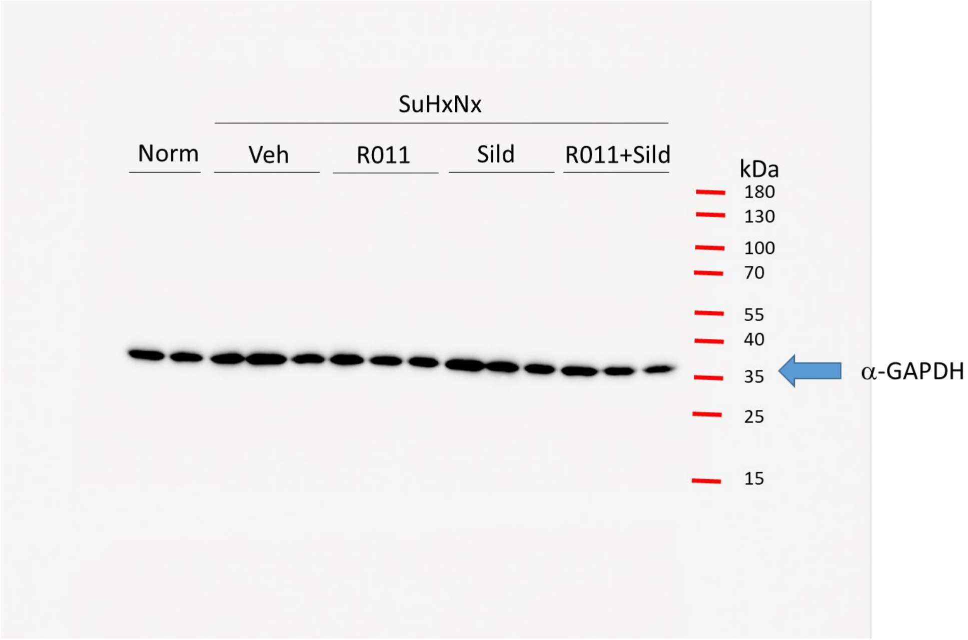

Supplemental Figure 9. (**A**) Uncropped pSmad2/3 immunoblot gel, (**B**) uncropped pSmad1/5/8 immunoblot gel, and (**C**) uncropped GAPDH immunoblot gel corresponding to the composite cropped image displayed in Supplemental Figure 6.

A

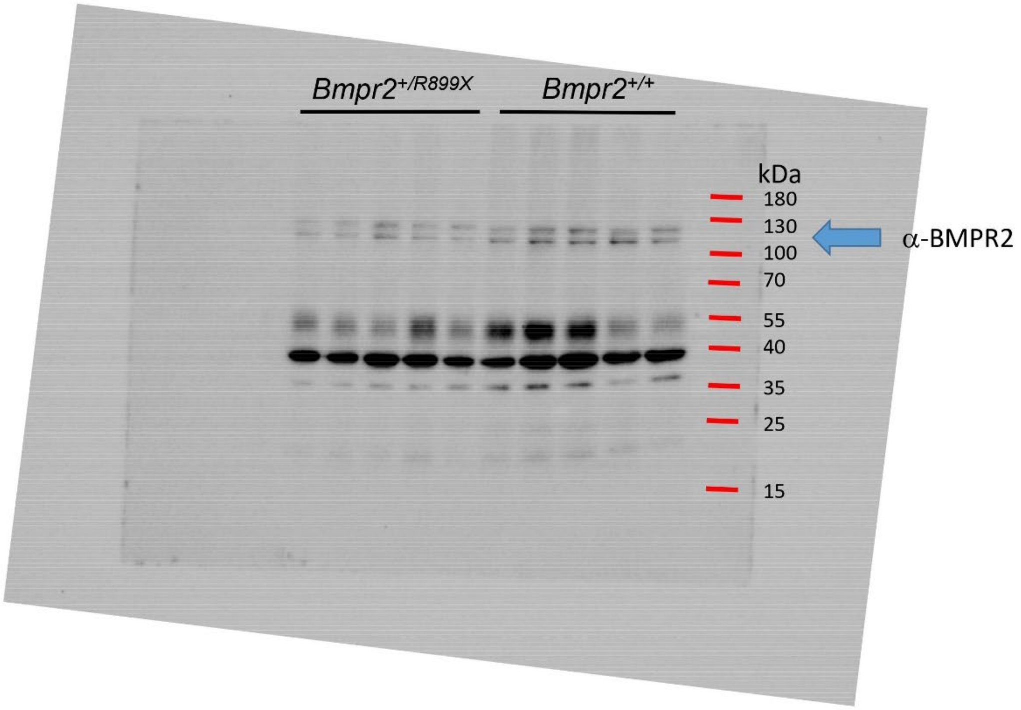

B

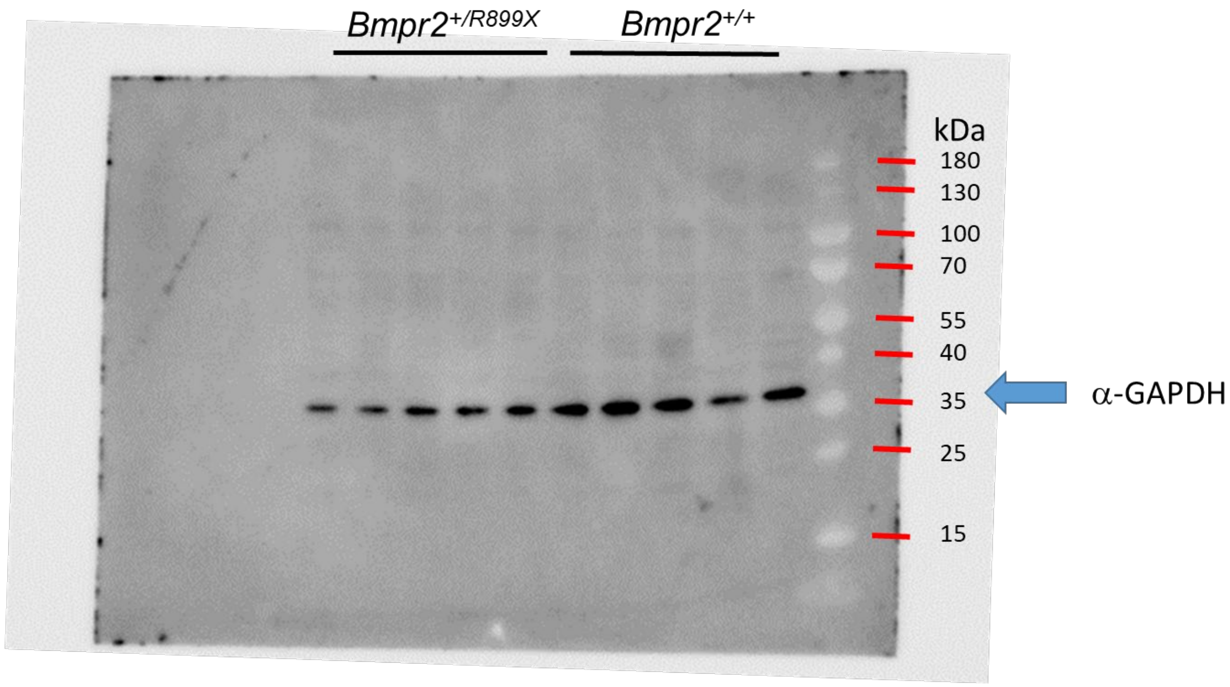

Supplemental Figure 10. (**A**) Uncropped BMPR**II** immunoblot gel and (**B**) uncropped GAPDH immunoblot gel corresponding to the composite cropped image displayed in Supplemental Figure 7.
